# Impact of feature selection methods and subgroup factors on prognostic analysis with CT-based radiomics in non-small cell lung cancer patients

**DOI:** 10.1186/s13014-021-01810-9

**Published:** 2021-04-30

**Authors:** Yuto Sugai, Noriyuki Kadoya, Shohei Tanaka, Shunpei Tanabe, Mariko Umeda, Takaya Yamamoto, Kazuya Takeda, Suguru Dobashi, Haruna Ohashi, Ken Takeda, Keiichi Jingu

**Affiliations:** 1 Department of Radiation Oncology, Tohoku University Graduate School of Medicine, 1-1 Seiryo-machi, Aoba-ku, Sendai, 980-8574 Japan; 2 Department of Radiological Technology, School of Health Sciences, Faculty of Medicine, Tohoku University, Sendai, Japan

**Keywords:** Radiomics, Prognosis prediction, Feature selection, Subgroup analysis, Lung cancer

## Abstract

**Background:**

Radiomics is a new technology to noninvasively predict survival prognosis with quantitative features extracted from medical images. Most radiomics-based prognostic studies of non-small-cell lung cancer (NSCLC) patients have used mixed datasets of different subgroups. Therefore, we investigated the radiomics-based survival prediction of NSCLC patients by focusing on subgroups with identical characteristics.

**Methods:**

A total of 304 NSCLC (Stages I–IV) patients treated with radiotherapy in our hospital were used. We extracted 107 radiomic features (i.e., 14 shape features, 18 first-order statistical features, and 75 texture features) from the gross tumor volume drawn on the free breathing planning computed tomography image. Three feature selection methods [i.e., test–retest and multiple segmentation (FS1), Pearson's correlation analysis (FS2), and a method that combined FS1 and FS2 (FS3)] were used to clarify how they affect survival prediction performance. Subgroup analysis for each histological subtype and each T stage applied the best selection method for the analysis of All data. We used a least absolute shrinkage and selection operator Cox regression model for all analyses and evaluated prognostic performance using the concordance-index (C-index) and the Kaplan–Meier method. For subgroup analysis, fivefold cross-validation was applied to ensure model reliability.

**Results:**

In the analysis of All data, the C-index for the test dataset is 0.62 (FS1), 0.63 (FS2), and 0.62 (FS3). The subgroup analysis indicated that the prediction model based on specific histological subtypes and T stages had a higher C-index for the test dataset than that based on All data (All data, 0.64 vs. SCC_all_, 060; ADC_all_, 0.69; T1, 0.68; T2, 0.65; T3, 0.66; T4, 0.70). In addition, the prediction models unified for each T stage in histological subtype showed a different trend in the C-index for the test dataset between ADC-related and SCC-related models (ADC_T1_–ADC_T4_, 0.72–0.83; SCC_T1_–SCC_T4_, 0.58–0.71).

**Conclusions:**

Our results showed that feature selection methods moderately affected the survival prediction performance. In addition, prediction models based on specific subgroups may improve the prediction performance. These results may prove useful for determining the optimal radiomics-based predication model.

**Supplementary Information:**

The online version contains supplementary material available at 10.1186/s13014-021-01810-9.

## Introduction

Non-small-cell lung cancer (NSCLC) accounts for approximately 85% of lung cancers [1], which makes it the leading cause of cancer mortality worldwide [2]. Although treatment decisions and prognostic of lung cancer have significantly improved over the years, a parallel improvement in terms of global survival rate has lagged [3]. Currently, the tumor-node-metastasis (TNM) staging system is the most reliable prognostic factor for lung cancer [4]. However, survival rates may vary between patients included in the same disease stage [5]. Therefore, new prognostic approaches are urgently needed to achieve a personalized medical treatment to improve disease outcome [6]. Personalized cancer treatment is now largely based on medical imaging [7] because it offers the advantages of being noninvasive, reproducible, and relatively easy to implement in clinical practice [8]. Of particular interest is the work of Aerts et al. [9], who showed that features extracted from computed tomography (CT) may be useful for predicting the outcome of NSCLC patients.

Radiomics is a high-throughput technique to quantify phenotypic features in medical images [9, 10]. These features may help predict survival prognosis, preoperative distant metastasis, and histological subtype classification [9, 11, 12]. In recent years, there have been several reports showing high accuracy of radiomics in predicting histological classification of NSCLC. Liu et al. [13] established a multi-subtype classification model for the four major histological subtypes of NSCLC [i.e., squamous cell carcinoma (SCC), adenocarcinoma (ADC), large cell carcinoma (LCC), and not otherwise specified (NOS)] and investigated their classification performance and generalization ability. They showed an average classification accuracy of 0.89 on the training set and a classification accuracy of 0.86 on the test set. Zhu et al. [14] sought to distinguish SCC and ADC based on a radiomic signature. Their results showed an area under the curve (AUC) of 0.905 for the training cohort and an AUC of 0.893 for the validation cohort, which indicated good performance of the radiomic signature in distinguishing between ADC and SCC. These studies that classified the histological subtypes of NSCLC showed that texture features representing heterogeneity were essential in classifying each subtype. This is synonymous with the fact that each subtype has a different trend in radiomic features (especially texture features), i.e., the way the tumor looks in the CT image. In addition to differences in radiomic features, significant differences in prognosis have been reported for each histological subtype [15, 16]. Abel et al. showed that, compared to ADC, SCC was highly associated with local recurrence rates and was an independent negative predictor of overall survival [15]. Thus, histological subtypes vary widely in all aspects, including phenotype, heterogeneity, and prognosis.

Many radiomics-based prognostic studies of NSCLC patients have used machine learning with mixed datasets of various subgroups [17–19]. In contrast, few studies focused on subgroups for prognostic analysis. Chaddad et al. [20] performed a prognostic analysis in each NSCLC subgroup (i.e., histological subtypes, TNM stages, and clinical stages) and obtained an AUC of 0.757, 0.703, 0.703, and 0.762 for LCC, T2, N0, and Stage I groups, respectively. In the abovementioned study, there was minimum evaluation of the improvement in prediction performance by subgroup analysis because the analysis group was too limited. Yang et al. [21] developed and validated a radiomic method by integrating tumor and lymph node radiomics for the preoperative prediction of lymph node status in gastric cancer. They performed validation using subgroups in the test dataset and showed an improvement in prediction performance compared to the validation using the whole dataset. In the abovementioned study, there was minimal subgroup analysis because the training model itself used the whole dataset rather than subgroups.

As described above, there are only studies with minimum evaluation of subgroup analysis, even though the trends of radiomic features differ in each subgroup because of differences in phenotype and heterogeneity. We hypothesized that proper training and validation using the NSCLC subgroup dataset would lead to high improvement in prognostic performance because it would eliminate differences in trends of radiomic features. Therefore, this study investigated the radiomics-based survival prediction for subgroup datasets with specific histological subtypes and T stages of NSCLC patients.

## Materials and methods

### Patient population and image acquisition

The dataset in this study included a total of 384 patients treated with radiotherapy for NSCLC in our hospital from January 2010 to October 2017. A subset of this dataset was classified by radiation oncologists with respect to tumor (T), lymph node (N), and metastasis (M) and classified into four standard clinical stages. In addition, the histological subtype of each patient was identified (i.e., SCC, ADC, LCC, and NOS). Further, all patients were labeled in terms of “survival,” “death,” and survival time in days from scan to death or to the date of last visit (i.e., censored). GE Light Speed RT16 (GE Medical Systems, Waukesha, WI, USA) was used at the resolution of 512 × 512 × slices to acquire CT images under free breathing, with the number of slices varying between subjects. The in-plane pixel size of the images was 0.703–1.172 × 0.703–1.172 mm^2^, and the slice thickness was 2.5 mm. For each scan, the gross tumor volume (GTV) was manually delineated by a radiation oncologist. Table 1 shows the demographic information of each patient. The dataset used in this study includes multiple clinical stages and chemotherapy status. This was done to reduce the bias outside of specific subgroup to evaluate the prognostic performance of specific subgroups in the subgroup analysis described below. The following patients were excluded: patients without a histological subtype report (n = 27) and with a lack of GTV contours (n = 53). The data for the remaining 304 patients were divided into training and test datasets using stratified sampling at the ratio of 80% and 20%, respectively, while maintaining a constant ratio of deaths to surviving patients.

**Table 1:** Patient characteristics

Characteristics	Total (n = 304)
Age (years: median [range])	71 [22–93]
*Gender*	
Male	252 (83%)
Female	52 (17%)
*Histological subtype*	
Squamous cell carcinoma	135 (44%)
Adenocarcinoma	149 (49%)
Large cell carcinoma	7 (2%)
Not otherwise specified	13 (4%)
*T stage*	
0	1 (0%)
1	93 (31%)
2	96 (32%)
3	49 (16%)
4	55 (18%)
*N stage*	
0	110 (36%)
1	37 (12%)
2	103 (34%)
3	47 (15%)
*M stage*	
0	253 (83%)
1	42 (14%)
*Clinical stage*	
I	83 (27%)
II	25 (8%)
III	146 (48%)
IV	41 (13%)
*Chemotherapy status*	
Yes	140 (46%)
No	164 (54%)
Survival time (days: median [range])	598 [1–3364]
*Survival status*	
Survival	126 (41%)
Death	178 (59%)

### Overall scheme

**Fig. 1:**
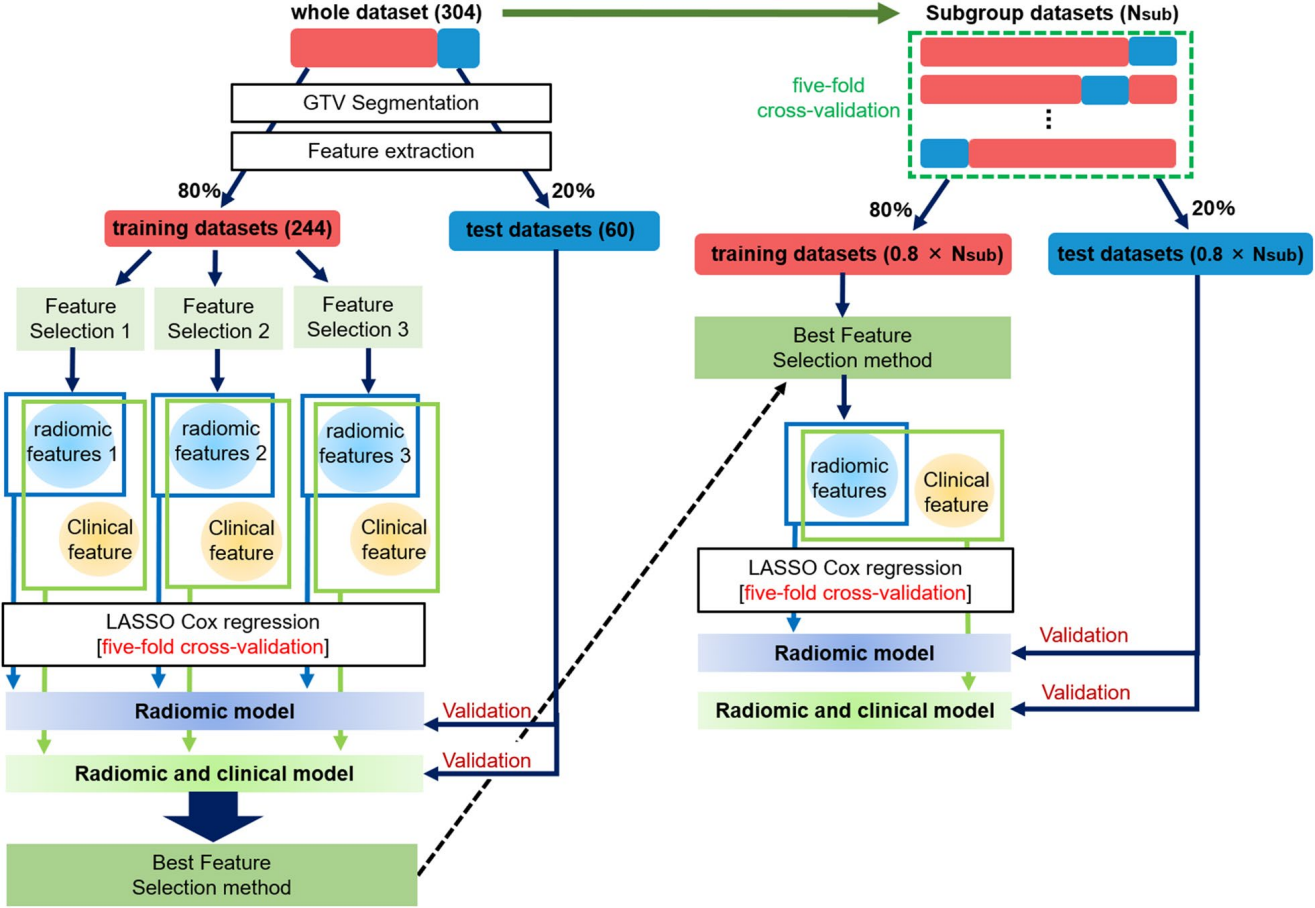
Overall scheme

Figure 1 shows the overall scheme of this study. First, we extracted a number of radiomic features from GTV segmentation for the whole dataset. Then, we divided the whole dataset into training and test datasets and applied three independent feature selection methods to the training dataset. Next, a least absolute shrinkage and selection operator (LASSO) Cox regression model was used to construct the model of radiomic features alone (radiomic model) and the model combining radiomic and clinical features (combined model). A test dataset was adapted to the constructed model, and the concordance-index (C-index) was used to evaluate the prognostic performance. Next, specific subgroup datasets were created from the whole dataset. In the subgroup analysis, a fivefold cross-validation was applied in model validation. The training dataset was subjected to the feature selection method that showed the best performance in the analysis for All data. As with the analysis for All data, LASSO Cox regression model was used to construct the radiomic and combined models, and the model performance was validated by applying the test dataset to the constructed model.

### Feature extraction

PyRadiomics [22] on 3D Slicer was used to extract radiomic features from GTV, which was resampled to 1 × 1 × 1 mm^3^. A total of 107 features were extracted for each patient (Additional file 1: Supplementary A), which includes 14 shape features, 18 first-order statistical features, and 75 texture features. The shape feature quantified the diameter and volume of the region of interest (ROI) and the degree of irregularity. The first-order statistical feature was used to create a histogram of pixel values and define features with respect to that histogram. The texture feature served to convert the relationships between pixel values into matrix to measure image uniformity and heterogeneity. In addition, the texture feature included gray-level co-occurrence matrix (GLCM, n = 24), gray-level dependence matrix (GLDM, n = 14), gray-level run length matrix (GLRLM, n = 16), gray-level size zone matrix (GLSZM, n = 16), and neighborhood gray tone difference matrix (NGTDM, n = 5). For the GLCM feature, the bin width was set to 25 Hounsfield units. The radiomic features in PyRadiomics were based on the Image Biomarker Standardization Initiative, which established the validated definitions and benchmarks of the features, except for four features: shape_Maximum2DDiameterSlice, shape_Maximum2DDiameterColumn, shape_Maximum2DDiameterRow, and first-order_TotalEnergy [23].

### Feature selection

The 107 radiomic features extracted from GTV were reduced using three independent feature selection methods. These three methods were applied to the training dataset in the whole dataset. This approach was used to determine the best feature selection methods in terms of prognostic performance. Then, the selection method that showed the best performance was used in the subgroup analysis.

Feature Selection 1 (FS1) selects only robust features using test–retest and multiple segmentation [9, 24]. The test–retest method uses a dataset created by Zhao et al. to evaluate the variability of tumor unidimensional, bidimensional, and volumetric measurements on same-day repeat CT scans [25]. This dataset can be downloaded from the publicly available online Reference Image Database to Evaluate Therapy Response (RIDER) test–retest dataset in the Cancer Imaging Archive (TCIA); this dataset consists of chest CT images of 32 patients that have been acquired twice at 15-min interval [26]. The test–retest method applies a radiomic analysis of tumors to two images of each patient and excludes features that significantly change over this short time as being less robust. The concordance correlation coefficient (CCC) served to evaluate the agreement between the values of two features, and feature selection was performed with CCC > 0.85 [24, 27, 28]. The multiple segmentation method uses a dataset created by van Baardwijk et al. to investigate whether auto-delineation reduces the interobserver variability compared to manual PET-CT–based GTV delineation [29]. This dataset can be downloaded from the publicly available online Quantitative Imaging Network multisite collection of lung CT data with nodule segmentations and RIDER data; this dataset consists of chest CT images of 20 patients that have been contoured by multiple physicians [26]. The multiple segmentation method uses a radiomic analysis of multiple ROIs of each patient and excludes features that vary significantly with small differences in contouring as being less robust. In addition, the intraclass correlation coefficient (ICC) served to evaluate the agreement between the values of multiple features, and feature selection was performed with ICC > 0.8 [24, 30]. In this study, we used 23 radiomic features selected in a previous study by Kadoya et al., who already performed test–retest and multiple segmentation using the abovementioned dataset and method [24].

Feature Selection 2 (FS2) excludes one of the correlated features from the analysis as redundant based on the correlation coefficients calculated by Pearson's correlation analysis for all features [21, 31]. An absolute value of the correlation coefficient of 0.8 or greater was the threshold to indicate strong correlation between two features [31, 32].

Feature Selection 3 (FS3) combines FS1 and FS2 [33, 34]. After robust features are selected using test–retest and multiple segmentation, non-redundant features are selected using Pearson's correlation analysis with a threshold of 0.8.

FS1, FS2, and FS3 are commonly used as feature selection methods for prognostic studies based on radiomics [9, 21, 33, 34]; therefore, we decided to adopt these feature selection methods in this study. MATLAB R2020a was used for all selection methods. Additional file 1: Supplementary B–D summarizes the robust and/or non-redundant features selected by FS1, FS2, and FS3, which were 23, 28, and 9, respectively.

### Clinical predictors

As long as clinical predictors significantly affect prognosis [17, 21, 35], the most representative clinical predictors were added to the features used in this study. We used a total of eight clinical predictors, namely, gender, age, each TNM stage, clinical stage, histological subtype, and chemotherapy status [36–38].

### Construction of the LASSO Cox regression model

Two different models were constructed: a model of radiomic features alone using the selected features in FS1, FS2, and FS3 (radiomic model) and a model combining radiomic and clinical features using the selected features plus clinical predictors (combined model). The LASSO Cox regression model was used to construct the model to predict survival prognosis. This regression model has often been used for radiomic analysis [18, 39].

Depending on λ, which is the weight of the constraint term on the likelihood function, the LASSO operation shrinks all regression coefficients toward zero and zeros the coefficients of irrelevant features. Learning models strongly depend on λ, such that large λ simplifies the model, whereas small λ reduces the role of weights and causes overfitting. We applied a fivefold cross-validation to prevent model simplification and overfitting and to select optimal λ for the data. In the fivefold cross-validation, to obtain model parameters, the dataset used for training was randomly divided into five parts, four of which were used as training data, and the remaining one was used as validation data. A model optimized for each λ for the training data was applied to the validation data, and the square error of residuals between the validation data and the model was computed. This treatment was repeated five times, and the five resulting square errors calculated for each λ were averaged to determine optimal λ for the smallest mean square error. Rad scores were calculated from linear combinations of features with nonzero coefficients at optimal λ. The rad score is represented by the sum of the nonzero coefficient features weighted by their respective coefficients (β), as shown in Eq. (1).

1 $$\mathrm{Rad}\,\mathrm{score}= \sum_{i=1}^{n}{\beta }_{i}\cdot {feature}_{i}$$

### Statistical analysis

A Kaplan–Meier survival analysis served to evaluate the association between the rad score and survival. The median rad score calculated by Eq. (1) provided the threshold for dividing training dataset into high- and low-risk groups, and Kaplan–Meier curves were created for each risk group. Then, the log-rank test tested for significant differences between high- and low-risk groups. The C-index was used to evaluate the prognostic performance. The test dataset was applied to the rad score Eq. (1) and evaluated using Kaplan–Meier survival analysis and the C-index as well as the training dataset.

Statistical analysis was performed using the R software 3.6.1 (http://www.R-project.org), where the R packages “survival”, “glmnet”, and “survminer” used the LASSO Cox regression model. Statistical significance was set at *P* < 0.05.

### Subgroup analysis

The whole dataset containing 304 NSCLC patients was classified into histological subtypes and T stages to create the subgroup datasets. The histological subtypes SCC, ADC, LCC, and NOS contained 149, 135, 7, and 13 patients, respectively. However, the LCC and NOS subtypes were excluded from the analysis because they contained too few data to analyze. The T stages T0–T4 and TX contained 1, 93, 96, 49, 55, and 10 patients, respectively. Again, T0 and TX stages were excluded from the analysis group because of too few data. For further analysis by datasets with identical characteristics, the SCC and ADC subtypes were classified into T stages T1–T4. Altogether, a total of 14 groups were included in the subgroup analysis. The SCC and ADC subtypes that contained all T stages were denoted SCC_all_ and ADC_all_, respectively, and those were further classified into T stages T1–T4 and denoted SCC_T1_, SCC_T2_, SCC_T3_, SCC_T4_, ADC_T1_, ADC_T2_, ADC_T3_, and ADC_T4_, respectively. Additional file 1: Supplementary E lists the patient characteristics for each subgroup.

As shown in Fig. 1, a fivefold cross-validation was used to validate the constructed model in subgroup analysis. Each subgroup dataset was divided into five parts using stratified sampling, while maintaining a constant ratio of deaths to surviving patients; then, four parts were set as the training dataset and one part as the test dataset. Fivefold cross-validation was used to ensure reliability of the model constructed with subgroup datasets with a small number of data. In addition, cross-validation may remove redundancy in the constructed model because, unlike the bootstrap method, it divides the dataset without allowing duplication. The C-indexes of both the radiomic and combined models in the analysis for All data were averaged for each feature selection method, and the method that produced the highest C-index was applied to the training dataset. Similar to the analysis for All data, the LASSO Cox regression model was used to construct radiomic models and combined models, and the test dataset was applied to each model. The Kaplan–Meier survival analysis and C-index were used to evaluate prognostic performance of the constructed model. The C-index used in the evaluation is the average of C-indexes of the five models constructed by the fivefold cross-validation. To compare the results of the whole dataset and the subgroup dataset under the same conditions, the same validation method as for the subgroup analysis was applied to All data.

To increase the reliability of this study, we applied the same subgroup analysis to a publicly available dataset (Lung 1, NSCLC-Radiomics) on TCIA [26]. Supplemental M indicates patient characteristics for this dataset. Other detailed information can be found in the paper by Aerts et al. [9]. Similar to the process applied to our dataset, after extracting 107 radiomic features from GTV of each patient in the Lung 1 dataset, Pearson's correlation analysis was applied to the training dataset as feature selection. We constructed radiomic models using only selected radiomic features. We also constructed combined models by adding clinical features to the radiomic model. However, because the Lung 1 dataset did not contain information on the chemotherapy status, a total of seven clinical features were included, excluding the chemotherapy status. The analysis was limited to three groups, including All data (n = 287), SCC_all_ (n = 82), and ADC_all_ (n = 27), owing to the number of data in each subgroup dataset. We applied exactly the same methods of learning and evaluation as described above.

## Results

Table 2 shows the prognosis prediction performance when robust and/or non-redundant features are used in the analysis for All data. FS2 had the highest C-index of all selection methods in the training and test datasets for the radiomic model (0.64 and 0.61, respectively). Similarly, FS2 had the highest C-index of all selection methods in the training and test datasets for the combined model (0.65 and 0.63, respectively). Therefore, FS2 with Pearson's correlation analysis was applied for subgroup analysis.

**Table 2:** Prognosis prediction performance when robust and/or non-redundant features are used in the analysis for All data

Constructed model	Total number of features	Training dataset		Test dataset	
		C-index	Hazard ratio (95%CI)	C-index	Hazard ratio (95%CI)
*Radiomic model*					
FS1	23	0.63*	1.55 (1.30–1.85)	0.60	0.95 (0.82–1.10)
FS2	28	0.64*	3.96 (2.43–6.45)	0.61*	1.87 (0.88–3.99)
FS3	9	0.62*	1.84 (0.17–2.19)	0.60*	1.06 (0.01–2.08)
*Combined model*					
FS1 + clinical	31	0.64*	2.22 (0.58–3.22)	0.62*	1.20 (0.45–2.87)
FS2 + clinical	36	0.65*	4.75 (2.99–7.56)	0.63*	2.24 (1.13–4.36)
FS3 + clinical	17	0.64*	2.62 (0.90–3.96)	0.62	0.94 (0.19–2.32)

*FS* Feature Selection, *CI* confidence interval

FS1: a method to select only robust features using test–retest and multiple segmentation

FS2: a method of excluding one of the correlated features from the analysis as redundant based on the correlation coefficients calculated by Pearson's correlation analysis for all features

FS3: a method that combined FS1 and FS2

*P value < 0.05

Table 3 shows the prognostic performance for each subgroup, and Additional file 1: Supplementary F–G show the prognostic performance of radiomic and combined models in each subgroup with fivefold cross-validation. In addition, Additional file 1: Supplementary I–J show Kaplan–Meier curves when divided into low- and high-risk groups based on the rad score in the radiomic and combined models for each subgroup. To avoid complications, the case when it was closest to the mean C-index of the test dataset among the fivefold cross-validation is shown. In the analysis of histological subtypes and T stages, both the radiomic and combined models produced higher C-indexes than did All data for all subgroups (except for the SCC_all_ group). In particular, the C-index of the test dataset in the radiomic model improved the most for the T1 group (0.62 ± 0.03 for All data vs. 0.66 ± 0.04 for the T1 group), and that in the combined model improved the most for the T4 group (0.64 ± 0.04 for All data vs. 0.70 ± 0.06 for the T4 group). Kaplan–Meier curves representing the relationship between the rad score and survival time also showed that patients in ADC_all_ and each T stage were significantly stratified between high and low rad score values compared to All data. The analysis of each T stage in the histological subtypes had different trends in the SCC and ADC groups. In the analysis of each T stage in the ADC group, both the radiomic and combined models produced higher C-indexes than the ADC_all_ group for all groups. In particular, the C-index of the test dataset in the radiomic model increased the most for the ADC_T3_ group (0.64 ± 0.02 for the ADC_all_ group vs. 0.81 ± 0.03 for the ADC_T3_ group), and that in the combined model increased the most for the ADC_T1_ group (0.69 ± 0.04 for the ADC_all_ group vs. 0.83 ± 0.04 for the ADC_T1_ group). Conversely, in the analysis of each T stage in the SCC group, both the radiomic and combined models produced considerably lower C-indexes in the test dataset of the SCC_T1_, SCC_T2_, and SCC_T3_ groups than of the SCC_all_ group. In addition, all T stage groups in SCC failed to stratify high and low rad score values in the Kaplan–Meier curves. In the analysis of all subgroups, the combined model showed a slightly or moderately higher C-index than did the radiomic model (Table 3).

**Table 3:** Prognosis prediction performance for each subgroup

Subgroup	Radiomic model		Combined model	
	Training, mean ± sd	Test, mean ± sd	Training, mean ± sd	Test, mean ± sd
All data (n = 304)	0.63 ± 0.01*	0.62 ± 0.03*	0.65 ± 0.01*	0.64 ± 0.04*
SCC_all_ (n = 135)	0.60 ± 0.03	0.59 ± 0.03	0.62 ± 0.04	0.60 ± 0.05
ADC_all_ (n = 149)	0.66 ± 0.02*	0.64 ± 0.02*	0.70 ± 0.02*	0.69 ± 0.04*
T1 (n = 93)	0.66 ± 0.03*	0.66 ± 0.04*	0.70 ± 0.02*	0.68 ± 0.03*
T2 (n = 96)	0.64 ± 0.03*	0.63 ± 0.05*	0.66 ± 0.02*	0.65 ± 0.02*
T3 (n = 49)	0.68 ± 0.02*	0.65 ± 0.03*	0.68 ± 0.04*	0.66 ± 0.06*
T4 (n = 55)	0.65 ± 0.02*	0.63 ± 0.04*	0.72 ± 0.02*	0.70 ± 0.06*
SCC_T1_ (n = 40)	0.59 ± 0.05	0.57 ± 0.05	0.61 ± 0.03	0.58 ± 0.04
SCC_T2_ (n = 41)	0.57 ± 0.04	0.55 ± 0.04	0.61 ± 0.03	0.59 ± 0.05
SCC_T3_ (n = 26)	0.69 ± 0.05	0.58 ± 0.04	0.71 ± 0.08	0.59 ± 0.04
SCC_T4_ (n = 25)	0.71 ± 0.02	0.71 ± 0.04	0.74 ± 0.05*	0.71 ± 0.03
ADC_T1_ (n = 46)	0.78 ± 0.02*	0.75 ± 0.05*	0.84 ± 0.03*	0.83 ± 0.04*
ADC_T2_ (n = 48)	0.70 ± 0.02*	0.68 ± 0.05*	0.72 ± 0.01*	0.72 ± 0.05*
ADC_T3_ (n = 20)	0.83 ± 0.04*	0.81 ± 0.03*	0.83 ± 0.04*	0.81 ± 0.02*
ADC_T4_ (n = 27)	0.71 ± 0.05*	0.70 ± 0.05*	0.75 ± 0.03*	0.73 ± 0.02*

*SCC* squamous cell carcinoma, *ADC* adenocarcinoma, *sd* standard deviation

*P value < 0.05

**Fig. 2:**
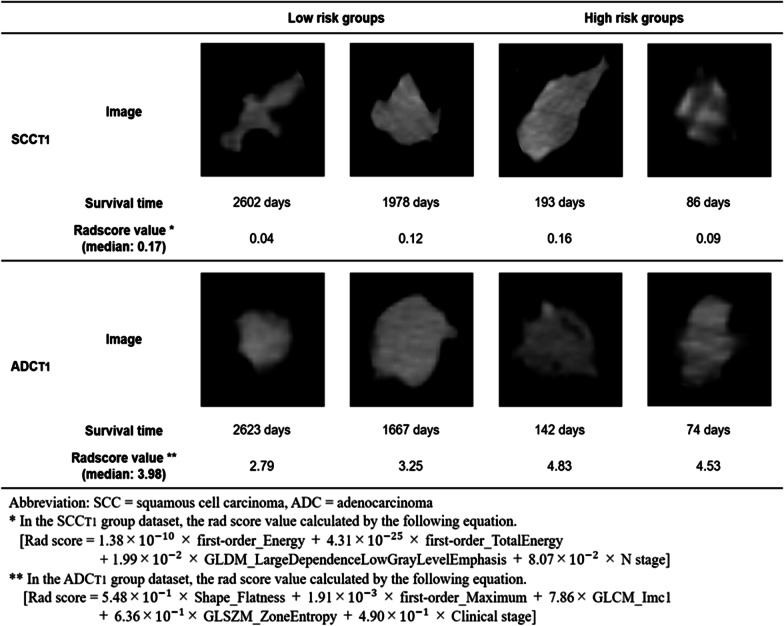
Representative cases to illustrate the difference in heterogeneity between ADC and SCC

Figure 2 shows representative cases to illustrate the difference in heterogeneity between ADC and SCC. As an example, we show CT images of GTV in two cases selected from the low- and high-risk groups of the ADC_T1_ and SCC_T1_ test datasets that showed the closest values to the mean C-index among the fivefold cross-validation. The abovementioned image shows the largest ROI slice in GTV. For the low-risk groups, survival time for the two cases was 2606 and 1978 days in the SCC_T1_ group and 2623 and 1667 days in the ADC_T1_ group. For the high-risk group, survival time was 193 and 86 days in the SCC_T1_ group and 142 and 74 days in the ADC_T1_ group. We also show the value of the rad score in the combined model for each subgroup. In both the SCC_T1_ and ADC_T1_ groups, the rad score equation includes the texture feature regarding uniformity. The abovementioned images show that, in the ADC_T1_ group, homogeneity is constant in the low-risk groups, whereas homogeneity is sparse in the high-risk groups. The rad score value is also below the median (3.98) in the low-risk groups and above the median in the high-risk groups. Conversely, in the SCC_T1_ group, the rad score value is below the median (0.17), despite the heterogeneity observed in the images in the high-risk group, which indicates a discrepancy between the images and radiomic features.

Similar results were observed in the subgroup analysis using the Lung 1 dataset (Additional file 1: Supplementary N–P). Compared to All data, the test datasets of both the radiomic and combined models produced a higher C-index for the ADC_all_ group and a lower C-index for the SCC_all_ group (Additional file 1: Supplementary N). The Kaplan–Meier curves, which show the case that was closest to the mean C-index of the test dataset among the fivefold cross-validation, showed that the ADC_all_ group was significantly stratified between high and low rad score values, whereas the SCC_all_ group was not (Additional file 1: Supplementary O). In addition, all three subgroups showed higher prognostic performance in the combined model than in the radiomic model.

## Discussion

Prognostic analysis of NSCLC patients using radiomics used mixed data with various subgroups [17–19]. In this study, we investigated the radiomics-based survival prediction for subgroup datasets with specific histological subtypes and T stages of NSCLC patients. The analysis of All data did not indicate high prognostic performance. However, the analysis of subgroups indicated better prognostic performance than did the analysis of All data. In particular, the analysis of each T stage in the ADC group produced a significant improvement in prognostic performance. This result suggests that the analysis of the NSCLC dataset by specific histological subtypes and T stages may significantly improve survival prediction.

This study applied three independent feature selection methods to All data to determine the best method in terms of prognostic performance. In the test dataset for both the radiomic and combined models, the highest prognostic performance is obtained when using FS2. Sun et al. [40] showed that Pearson's feature selection method (i.e., similar to FS2 in our method) in the Cox model produced the second highest C-index among the five selection methods. In addition, Leger et al. [41] also showed that the same Pearson's feature selection method in the Cox model produced the highest C-index among the 12 selection methods. These results are consistent with our result (i.e., FS2 had the highest C-index). Because the Cox model directly predicts the time to event with a simple regression equation, this model often produced overfitting [41]. Pearson's feature selection method is one of the filter-based methods, which can minimize overfitting by removing redundant feature interactions with high computational efficiency [42]. On the basis of these characteristics of the Cox model and Pearson's feature selection method, it can be explained that this selection method was the most useful method for prognosis prediction with the Cox model.

Previous studies, which validated the prediction performance by applying each subgroup dataset to the model trained with the whole dataset, have shown an improvement in prediction performance compared to applying the whole dataset [21, 43]. However, these studies have not constructed training models using subgroup datasets and performed only minimal subgroup analysis. Our study is the first study to construct both All data and subgroup models to perform prognostic analysis of NSCLC patients. The obtained results showed an improvement in prognostic performance in many subgroups (except for the SCC_all_, SCC_T1_, SCC_T2_, and SCC_T3_ groups) compared to All data. In particular, the best prognostic performance was achieved in the ADC_T1_ and ADC_T3_ groups (0.83 ± 0.04 and 0.81 ± 0.02, respectively). These are based on our hypothesis that high prognostic performance is produced by eliminating differences in trends of radiomic features among subgroups with different prognosis and heterogeneity. Therefore, the approach used in this study, in which the training model was constructed for each subgroup, may accurately reflect the characteristics of each group as a radiomic feature and may improve the performance of prognostic predictions.

Compared to that in All data, there was an improvement in prognostic performance in the ADC-related group, but there was a decrease in prognostic performance in some SCC-related groups. Two reasons may explain the degraded prognostic performance from the SCC-related groups. First, ADC occurs at a different site than does SCC. In general, ADC most commonly occurs at the peripheral of lung parenchyma. Conversely, SCC consists of mostly hilar-type lung cancers near the hilar area. In fact, the data used herein indicated that tumors occurred in the pulmonary hilar area in 22% of the SCC group, but in 8% of the ADC group. If a tumor is adjacent to the hilar area (i.e., contacts the main bronchus near the bronchial area), its boundaries may be difficult to determine when contouring. Second, there is a difference in the heterogeneity of ADC and SCC. Many studies have already reported that the heterogeneity difference between ADC and SCC is accurately represented as radiomic features [13, 14, 44]. However, this heterogeneity difference by histological subtype may have a significant impact on prognostic prediction. In other words, in the ADC-related group, the radiomic features may properly reflect tumor heterogeneity on the images, whereas in the SCC-related group, they do not, and may not have a clear difference in the heterogeneity separating the low- and high-risk groups.

Some studies have shown the potential clinical utility of the prognostic models based on radiomics analysis [9, 45]. This study aimed to achieve sufficient prognostic performance for clinical utility using an approach that focused on the prognostic analysis in subgroups with identical characteristics. Our results show relatively high prognostic performance in ADC-related subgroup datasets, which may bring us closer to potential clinical applications. However, there is a problem that must be addressed before future clinical applications are possible, i.e., the advent of therapies using immune checkpoint inhibitors and molecular targeted drugs. These therapies have considerably improved the prognosis of lung cancer patients [46]; thus, it is necessary to develop a prognostic model that accounts for these factors. Recently, high association with radiomics and potential for high prognosis prediction has been reported in a dataset of patients treated with these therapies [47–49]. A future challenge is to reveal whether the model can be adapted to data from patients who have been treated with the abovementioned treatments.

Finally, this study has several limitations. First, it considers the type of subgroups analyzed. Although excluded from this analysis owing to the considerable variation in the number of data between groups, the prognostic performance can be improved by unifying clinical stages that treatments and heterogeneity greatly varied between groups. Second, this study is based on a relatively small number of patients. Because the number of data for some subgroups is quite small, the results obtained herein require further validation using a study based on more data. Third, it considers the issue of contouring. Manual segmentation with a single oncologist was used in this study. Previous studies have reported that semi-automatic segmentation was useful owing to high reproducibility and reliability, although this method may have software dependence [50, 51].

## Conclusions

This study investigated the radiomics-based survival prediction for subgroup datasets with specific histological subtypes and T stages of NSCLC patients. Our results showed that the models based on ADC-related groups and each T stage group had a higher C-index than had the models based on All data. Therefore, the prognostic analysis of specific subgroups can be expected to significantly improve the performance of prognostics.

## Supplementary Information

**Additional file 1: Supplementary A.** List of radiomic features used in this study. **Supplementary B**: List of robust radiomic features. (FS1). **Supplementary C**: List of non-redundant radiomic features. (FS2). **Supplementary D**: List of robust and non-redundant radiomic features. (FS3). **Supplementary E**: Patient characteristics for each subgroup. **Supplementary F**: Prognostic performance of the radiomic model in each subgroup with five-fold cross-validation. **Supplementary G**: Prognostic performance of the combined model in each subgroup with five-fold cross-validation. **Supplementary H**: Feature selection using the LASSO Cox model in the radiomic and combined models for each subgroup. **Supplementary I**: Kaplan–Meier curves for low- and high-risk groups based on the rad score in the radiomic models for each subgroup. **Supplementary J**: Kaplan–Meier curves for low- and high-risk groups based on the rad score in the combined models for each subgroup. **Supplementary K**: For each analysis group, the features and their coefficients selected by the LASSO Cox regression model in the radiomic models. **Supplementary L**: For each analysis group, the features and their coefficients selected by the LASSO Cox regression model in the combined models. **Supplementary M**: Patient characteristics for the Lung 1 dataset. **Supplementary N**: Prognostic performance of the radiomic and combined models in each subgroup with five-fold cross-validation (Lung 1 dataset). **Supplementary O**: Kaplan–Meier curves for low- and high-risk groups based on the rad score in the radiomic and combined models for each subgroup (Lung 1 dataset). **Supplementary P**: For each analysis group, the features and their coefficients selected in the LASSO Cox regression model in the radiomic and combined models (Lung 1 dataset).

## Data Availability

There is no availability of these data, which were used under license for the current study and so are not publicly available.

## Abbreviations

NSCLC: Non-small-cell lung cancer
TNM: Tumor-node-metastasis
CT: Computed tomography
SCC: Squamous cell carcinoma
ADC: Adenocarcinoma
LCC: Large cell carcinoma
NOS: Not otherwise specified
AUC: Area under the curve
T: Tumor
N: Lymph node
M: Metastasis
GTV: Gross tumor volume
LASSO: Least absolute shrinkage and selection operator
C-index: Concordance-index
ROI: Region of interest
GLCM: Gray-level co-occurrence matrix
GLDM: Gray-level dependence matrix
GLRLM: Gray-level run length matrix
GLSZM: Gray-level size zone matrix
NGTDM: Neighborhood gray tone difference matrix
FS: Feature selection
TCIA: The Cancer Imaging Archive
RIDER: Reference Image Database to Evaluate Therapy Response
CCC: Concordance correlation coefficient
ICC: Intraclass correlation coefficient
ML: Machine learning
