# Supplementary material for: Impact of feature selection methods and subgroup factors on prognostic analysis with CT-based radiomics in non-small cell lung cancer patients
Source: Radiat Oncol. 2021 Apr 30;16:80. doi: 10.1186/s13014-021-01810-9 (PMC8086112; doi:10.1186/s13014-021-01810-9)
Supplement: Supplementary file 1 — Additional file 1: Supplementary A. List of radiomic features used in this study. Supplementary B: List of robust radiomic features. (FS1). Supplementary C: List of non-redundant radiomic features. (FS2). Supplementary D: List of robust and non-redundant radiomic features. (FS3). Supplementary E: Patient characteristics for each subgroup. Supplementary F: Prognostic performance of the radiomic model in each subgroup with five-fold cross-validation. Supplementary G: Prognostic performance of the combined model in each subgroup with five-fold cross-validation. Supplementary H: Feature selection using the LASSO Cox model in the radiomic and combined models for each subgroup. Supplementary I: Kaplan–Meier curves for low- and high-risk groups based on the rad score in the radiomic models for each subgroup. Supplementary J: Kaplan–Meier curves for low- and high-risk groups based on the rad score in the combined models for each subgroup. Supplementary K: For each analysis group, the features and their coefficients selected by the LASSO Cox regression model in the radiomic models. Supplementary L: For each analysis group, the features and their coefficients selected by the LASSO Cox regression model in the combined models. Supplementary M: Patient characteristics for the Lung 1 dataset. Supplementary N: Prognostic performance of the radiomic and combined models in each subgroup with five-fold cross-validation (Lung 1 dataset). Supplementary O: Kaplan–Meier curves for low- and high-risk groups based on the rad score in the radiomic and combined models for each subgroup (Lung 1 dataset). Supplementary P: For each analysis group, the features and their coefficients selected in the LASSO Cox regression model in the radiomic and combined models (Lung 1 dataset). [file 13014_2021_1810_MOESM1_ESM.pdf]

Supplementary A: List of radiomic features used in this study.

Shape	Elongation, Flatness, LeastAxisLength, MajorAxisLength, Maximum2DDiameterColumn, Maximum2DDiameterRow, Maximum2DDiameterSlice, Maximum3DDiameter, MeshVolume, MinorAxisLength, Sphericity, SurfaceArea, SurfaceVolumeRatio, VoxelVolume
first-order	10Percentile, 90Percentile, Energy, Entropy, InterquartileRange, Kurtosis, Maximum, MeanAbsoluteDeviation, Mean, Median, Minimum, Range, RobustMeanAbsoluteDeviation, RootMeanSquared, Skewness, TotalEnergy, Uniformity, Variance
GLCM	Autocorrelation, ClusterProminence, ClusterShade, ClusterTendency, Contrast, Correlation, DifferenceAverage, DifferenceEntropy, DifferenceVariance, Id, Idm, Idmn, Idn, Imc1, Imc2, InverseVariance, JointAverage, JointEnergy, JointEntropy, MCC, MaximumProbability, SumAverage, SumEntropy, SumSquares
GLDM	DependenceEntropy, DependenceNonUniformity, DependenceNonUniformityNormalized, DependenceVariance, GrayLevelNonUniformity, GrayLevelVariance, HighGrayLevelEmphasis, LargeDependenceEmphasis, LargeDependenceHighGrayLevelEmphasis, LargeDependenceLowGrayLevelEmphasis, LowGrayLevelEmphasis, SmallDependenceEmphasis, SmallDependenceHighGrayLevelEmphasis, SmallDependenceLowGrayLevelEmphasis
GLRLM	GrayLevelNonUniformity, GrayLevelNonUniformityNormalized, GrayLevelVariance, HighGrayLevelRunEmphasis, LongRunEmphasis, LongRunHighGrayLevelEmphasis, LongRunLowGrayLevelEmphasis, LowGrayLevelRunEmphasis, RunEntropy, RunLengthNonUniformity,

	RunLengthNonUniformityNormalized, RunPercentage, RunVariance, ShortRunEmphasis, ShortRunHighGrayLevelEmphasis, ShortRunLowGrayLevelEmphasis
GLSZM	GrayLevelNonUniformity, GrayLevelNonUniformityNormalized, GrayLevelVariance, HighGrayLevelZoneEmphasis, LargeAreaEmphasis, LargeAreaHighGrayLevelEmphasis, LargeAreaLowGrayLevelEmphasis, LowGrayLevelZoneEmphasis, SizeZoneNonUniformity, SizeZoneNonUniformityNormalized, SmallAreaEmphasis, SmallAreaHighGrayLevelEmphasis, SmallAreaLowGrayLevelEmphasis, ZoneEntropy, ZonePercentage, ZoneVariance
NGTDM	Busyness, Coarseness, Complexity, Contrast, Strength

Abbreviation: GLCM = gray-level co-occurrence matrix, GLDM = gray-level dependence matrix, GLRLM = gray-level run length matrix, GLSZM = gray-level size zone matrix, NGTDM = neighborhood gray tone difference matrix

Supplementary B: List of robust radiomic features. (FS1)

Shape	VoxelVolume, Maximum3DDiameter, MeshVolume, MajorAxisLength, LeastAxisLength, Maximum2DDiameterSlice, SurfaceArea, MinorAxisLength, Maximum2DDiameterRow
first-order	Range
GLCM	JointEntropy, DifferenceVariance
GLDM	DependenceNonUniformity, GrayLevelNonUniformity
GLRLM	GrayLevelNonUniformity, RunLengthNonUniformity
GLSZM	ZoneVariance, SizeZoneNonUniformity, GrayLevelNonUniformity, LargeAreaEmphasis, LargeAreaLowGrayLevelEmphasis
NGTDM	Complexity, Busyness

Abbreviation: GLCM = gray-level co-occurrence matrix, GLRLM = gray-level run length matrix, GLDM = gray-level dependence matrix, GLSZM = gray-level size zone matrix, NGTDM = neighborhood gray tone difference matrix

Supplementary C: List of non-redundant radiomic features. (FS2)

Shape	Flatness, MajorAxisLength, Sphericity, SurfaceVolumeRatio
first-order	10Percentile, 90Percentile, Energy, Kurtosis, Maximum, Minimum, Skewness, TotalEnergy
GLCM	ClusterShade, Correlation, Imc1, InverseVariance
GLDM	DependenceVarianc
GLSZM	GrayLevelNonUniformity, GrayLevelNonUniformityNormalized, GrayLevelVariance, LargeAreaEmphasis, LargeAreaLowGrayLevelEmphasis, SmallAreaEmphasis, SmallAreaLowGrayLevelEmphasis, ZoneEntropy
NGTDM	Coarseness, Complexity, Strength

Abbreviation: GLCM = gray-level co-occurrence matrix, GLDM = gray-level dependence matrix,
GLSZM = gray-level size zone matrix,
NGTDM = neighborhood gray tone difference matrix

Supplementary D: List of robust and non-redundant radiomic feature. (FS3)

Shape	MajorAxisLength
first-order	Range
GLCM	JointEnergy, DifferenceVariance
GLSZM	SizeZoneNonUniformity, LargeAreaEmphasis, LargeAreaLowGrayLevelEmphasis
NGTDM	Complexity, Busyness

Abbreviation: GLCM = gray-level co-occurrence matrix, GLSZM = gray-level size zone matrix,

NGTDM = neighborhood gray tone difference matrix

Supplementary E: Patient characteristics for each subgroup.

Subgroup	Male	Female	Age (years: median [range])	Survival time (days: median [range])	Status	
					survival	death
**All data (n = 304)**	252 (100%)	52 (%)	71 [22–93]	598 [1–3364]	126 (41%)	178 (59%)
**SCC_{all} (n = 135)**	128 (95%)	7 (5%)	71 [39–93]	526 [1–3364]	49 (36%)	86 (64%)
**ADC_{all} (n = 149)**	106 (71%)	43 (29%)	71 [42–88]	775 [8–3253]	70 (47%)	79 (53%)
**T1 (n = 93)**	75 (81%)	18 (19%)	76 [45–93]	820 [10–2875]	46 (49%)	47 (51%)
**T2 (n = 96)**	78 (81%)	18 (19%)	72.5 [22–88]	633 [9–3364]	34 (35%)	62 (65%)
**T3 (n = 49)**	41 (84%)	8 (16%)	68 [40–86]	505 [19–2770]	19 (39%)	30 (61%)
**T4 (n = 55)**	48 (87%)	7 (13%)	65 [39–88]	540 [38–3302]	22 (40%)	33 (60%)
**SCC_{T1} (n = 40)**	38 (95%)	2 (5%)	75.5 [48–93]	592 [10–2602]	15 (38%)	25 (62%)
**SCC_{T2} (n = 41)**	39 (95%)	2 (5%)	73 [53–85]	658 [9–3364]	14 (34%)	27 (66%)
**SCC_{T3} (n = 26)**	25 (96%)	1 (4%)	68 [53–86]	513 [19–2632]	10 (38%)	16 (62%)
**SCC_{T4} (n = 25)**	23 (92%)	2 (8%)	67 [39–88]	476 [43–3302]	10 (40%)	15 (60%)
**ADC_{T1} (n = 46)**	30 (65%)	16 (35%)	75.5 [45–87]	1001 [13–2840]	29 (63%)	17 (37%)
**ADC_{T2} (n = 48)**	33 (69%)	15 (31%)	72.5 [45–88]	592 [23–2807]	16 (33%)	32 (67%)
**ADC_{T3} (n = 20)**	14 (70%)	6 (30%)	69 [53–83]	513 [22–2770]	9 (45%)	11 (55%)
**ADC_{T4} (n = 27)**	22 (81%)	5 (19%)	65 [42–84]	786 [38–3253]	11 (41%)	16 (59%)

Abbreviation: SCC = squamous cell carcinoma, ADC = adenocarcinoma

Supplementary F: Prognostic performance of the radiomic model in each subgroup with five-fold cross-validation.

Subgroup		1	2	3	4	5	Overall
**All data (n = 304)**	training	0.63	0.63	**0.63**	0.61	0.63	0.63 ± 0.01
	test	0.57	0.64	**0.62**	0.66	0.62	0.62 ± 0.03
**SCC_{all} (n = 135)**	training	**0.59**	0.59	0.58	0.66	0.58	0.60 ± 0.03
	test	**0.57**	0.62	0.61	0.61	0.53	0.59 ± 0.03
**ADC_{all} (n = 149)**	training	0.62	0.68	0.67	**0.66**	0.65	0.66 ± 0.02
	test	0.61	0.64	0.65	**0.64**	0.66	0.64 ± 0.02
**T1 (n = 93)**	training	**0.65**	0.61	0.68	0.69	0.69	0.66 ± 0.03
	test	**0.65**	0.71	0.63	0.69	0.60	0.66 ± 0.04
**T2 (n = 96)**	training	0.59	0.67	0.65	0.65	**0.66**	0.64 ± 0.03
	test	0.56	0.65	0.58	0.70	**0.65**	0.63 ± 0.05
**T3 (n = 49)**	training	**0.71**	0.68	0.66	0.65	0.69	0.68 ± 0.02
	test	**0.65**	0.61	0.67	0.61	0.70	0.65 ± 0.03
**T4 (n = 55)**	training	0.67	0.66	0.61	0.64	**0.65**	0.65 ± 0.02
	test	0.69	0.61	0.65	0.58	**0.64**	0.63 ± 0.04
**SCC_{T1} (n = 40)**	training	**0.59**	0.66	0.55	0.65	0.52	0.59 ± 0.05
	test	**0.56**	0.63	0.62	0.53	0.50	0.57 ± 0.05

**SCC_{T2} (n = 41)**	training	0.60	0.60	0.53	0.50	**0.61**	0.57 ± 0.04
	test	0.52	0.53	0.62	0.53	**0.55**	0.55 ± 0.04
**SCC_{T3} (n = 26)**	training	**0.67**	0.75	0.74	0.65	0.64	0.69 ± 0.05
	test	**0.59**	0.63	0.51	0.59	0.56	0.58 ± 0.04
**SCC_{T4} (n = 25)**	training	0.74	**0.70**	0.70	0.73	0.69	0.71 ± 0.02
	test	0.71	**0.71**	0.68	0.78	0.67	0.71 ± 0.04
**ADC_{T1} (n = 46)**	training	**0.76**	0.81	0.79	0.76	0.76	0.78 ± 0.02
	test	**0.75**	0.72	0.69	0.85	0.76	0.75 ± 0.05
**ADC_{T2} (n = 48)**	training	0.71	0.71	0.67	**0.68**	0.74	0.70 ± 0.02
	test	0.66	0.78	0.64	**0.68**	0.65	0.68 ± 0.05
**ADC_{T3} (n = 20)**	training	0.85	**0.81**	0.90	0.79	0.79	0.83 ± 0.04
	test	0.83	**0.80**	0.80	0.75	0.85	0.81 ± 0.03
**ADC_{T4} (n = 27)**	training	0.67	0.72	0.66	0.79	**0.73**	0.71 ± 0.05
	test	0.67	0.71	0.66	0.77	**0.70**	0.70 ± 0.04

Abbreviation: SCC = squamous cell carcinoma, ADC = adenocarcinoma

Numbers written in bold text: Case closest to the average C-index of the test dataset among the five-fold cross-validation

Supplementary G: Prognostic performance of the combined model in each subgroup with five-fold cross-validation.

Subgroup		1	2	3	4	5	Overall
All data (n = 304)	training	**0.66**	0.65	0.66	0.64	0.65	0.65 ± 0.01
	test	**0.64**	0.69	0.62	0.68	0.58	0.64 ± 0.04
SCC_{all} (n = 135)	training	0.62	0.69	**0.62**	0.56	0.61	0.62 ± 0.04
	test	0.64	0.54	**0.59**	0.55	0.66	0.60 ± 0.05
ADC_{all} (n = 149)	training	0.68	0.69	0.70	0.73	**0.70**	0.70 ± 0.02
	test	0.68	0.76	0.70	0.62	**0.69**	0.69 ± 0.04
T1 (n = 93)	training	0.72	0.69	0.72	0.67	**0.69**	0.70 ± 0.02
	test	0.65	0.74	0.67	0.66	**0.69**	0.68 ± 0.03
T2 (n = 96)	training	0.66	0.63	0.65	**0.69**	0.69	0.66 ± 0.02
	test	0.69	0.63	0.63	**0.65**	0.63	0.65 ± 0.02
T3 (n = 49)	training	0.65	0.75	0.64	**0.70**	0.67	0.68 ± 0.04
	test	0.71	0.73	0.57	**0.69**	0.60	0.66 ± 0.06
T4 (n = 55)	training	**0.72**	0.73	0.74	0.74	0.68	0.72 ± 0.02
	test	**0.69**	0.80	0.63	0.73	0.66	0.70 ± 0.06
SCC_{T1} (n = 40)	training	0.57	0.66	0.63	0.60	**0.61**	0.61 ± 0.03
	test	0.60	0.64	0.56	0.52	**0.59**	0.58 ± 0.04

SCC_{T2} (n = 41)	training	**0.61**	0.67	0.58	0.61	0.60	0.61 ± 0.03
	test	**0.59**	0.60	0.53	0.68	0.55	0.59 ± 0.05
SCC_{T3} (n = 26)	training	0.63	0.81	**0.78**	0.61	0.72	0.71 ± 0.08
	test	0.62	0.65	**0.60**	0.53	0.55	0.59 ± 0.04
SCC_{T4} (n = 25)	training	0.80	0.73	0.79	**0.72**	0.66	0.74 ± 0.05
	test	0.72	0.75	0.73	**0.72**	0.65	0.71 ± 0.03
ADC_{T1} (n = 46)	training	0.88	0.80	0.81	0.85	**0.84**	0.84 ± 0.03
	test	0.87	0.87	0.77	0.80	**0.83**	0.83 ± 0.04
ADC_{T2} (n = 48)	training	0.71	0.72	0.71	0.72	**0.75**	0.72 ± 0.01
	test	0.65	0.79	0.74	0.67	**0.73**	0.72 ± 0.05
ADC_{T3} (n = 20)	training	0.88	0.82	**0.81**	0.88	0.78	0.83 ± 0.04
	test	0.83	0.79	**0.80**	0.85	0.79	0.81 ± 0.02
ADC_{T4} (n = 27)	training	0.73	0.81	0.73	**0.74**	0.73	0.75 ± 0.03
	test	0.72	0.76	0.71	**0.73**	0.75	0.73 ± 0.02

Abbreviation: SCC = squamous cell carcinoma, ADC = adenocarcinoma

Numbers written in bold text: Case closest to the average C-index of the test dataset among the five-fold cross-validation

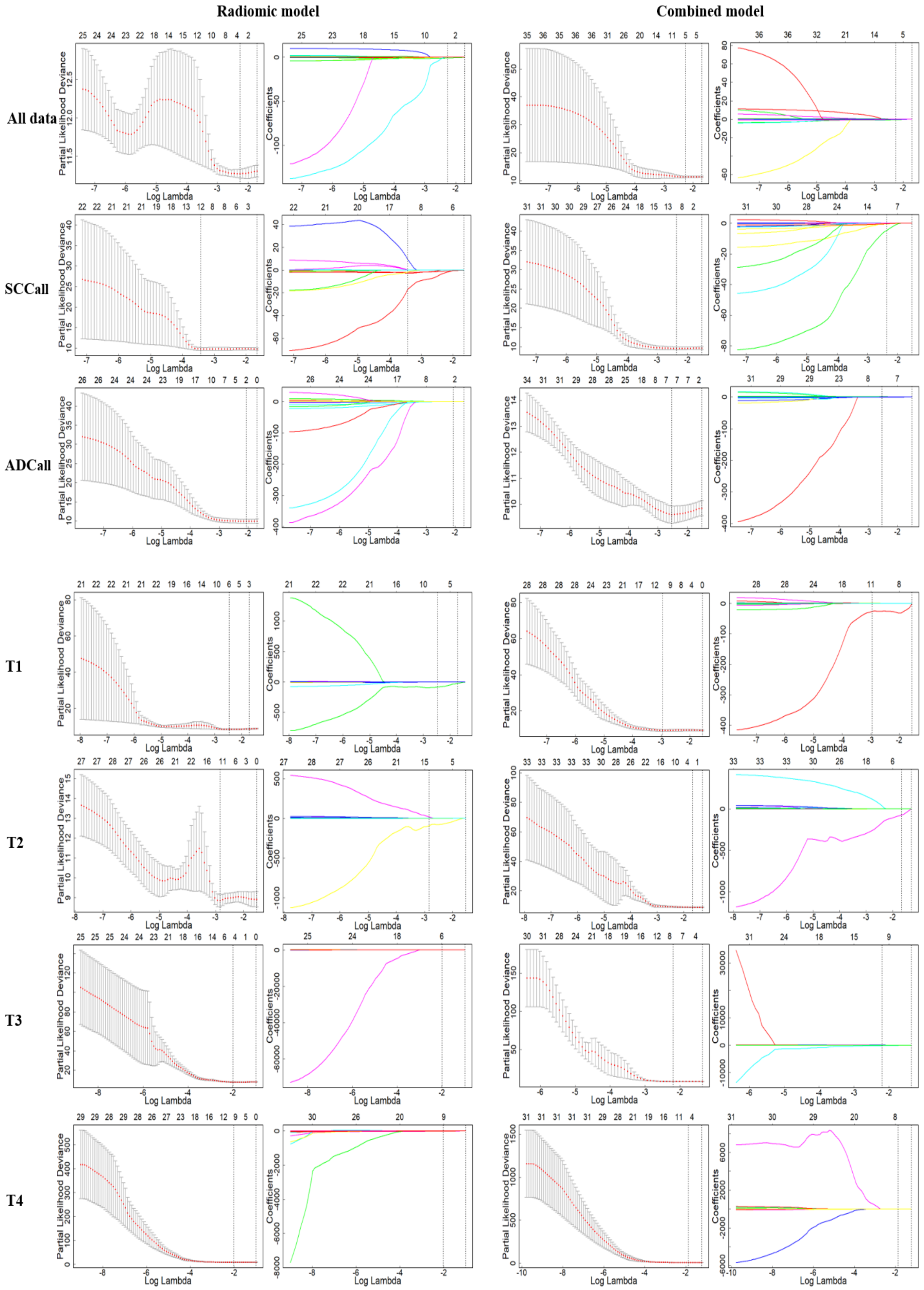
Radiomic model
Combined model
All data
SCCall
ADCall
T1
T2
T3
T4
Partial Likelihood Deviance
Coefficients
Log Lambda

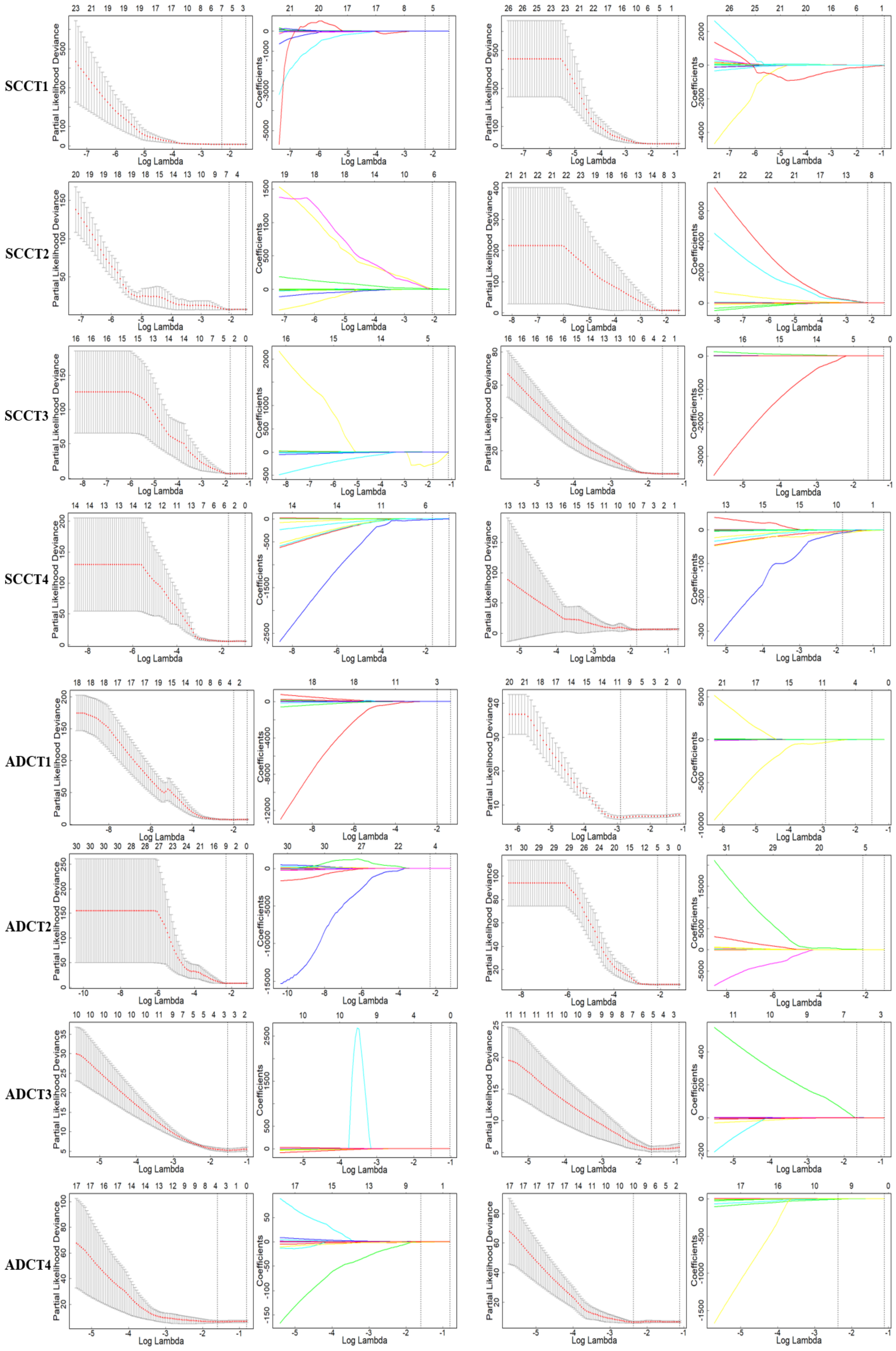
SCCT1
SCCT2
SCCT3
SCCT4
ADCT1
ADCT2
ADCT3
ADCT4
Partial Likelihood Deviance
Coefficients
Log Lambda

Supplementary H: Feature selection using the LASSO Cox model in the radiomic and combined models for each subgroup. To avoid complications, the case when it was closest to the mean C-index of the test dataset among the five-fold cross-validation is shown. λ with the lowest partial likelihood deviance was selected as optimal λ by five-fold cross-validation (each left figure). Lasso coefficient profile for each λ of the radiomic features (each right figure). The dotted vertical lines correspond to optimal lambda value according to the 1 standard error criteria and the minimum criteria.

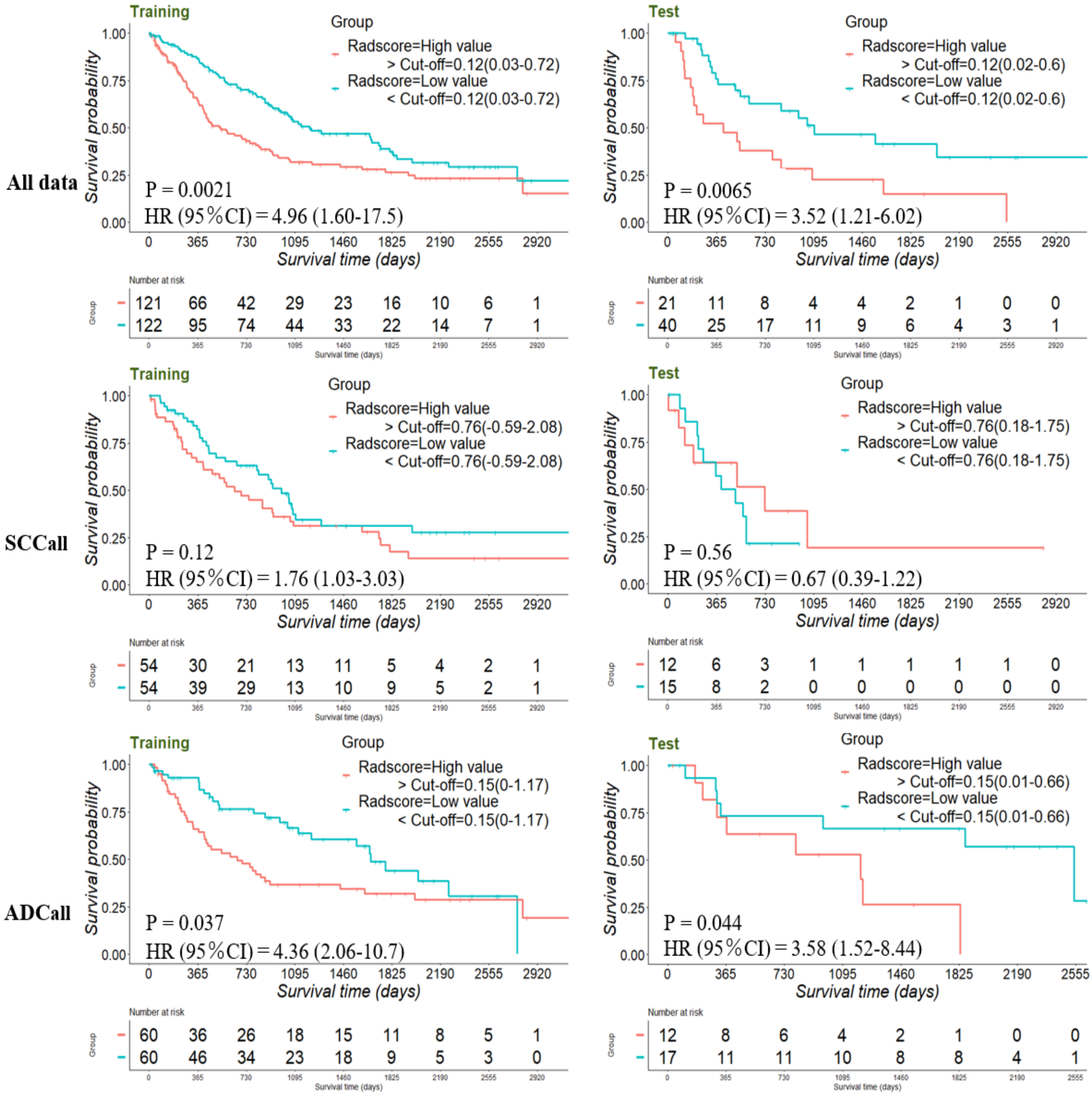
All data
SCCall
ADCall
Training
Test
Group
Radscore=High value
> Cut-off=0.12(0.03-0.72)
Radscore=Low value
< Cut-off=0.12(0.03-0.72)
P = 0.0021
HR (95%CI) = 4.96 (1.60-17.5)
> Cut-off=0.12(0.02-0.6)
< Cut-off=0.12(0.02-0.6)
P = 0.0065
HR (95%CI) = 3.52 (1.21-6.02)
> Cut-off=0.76(-0.59-2.08)
< Cut-off=0.76(-0.59-2.08)
P = 0.12
HR (95%CI) = 1.76 (1.03-3.03)
> Cut-off=0.76(0.18-1.75)
< Cut-off=0.76(0.18-1.75)
P = 0.56
HR (95%CI) = 0.67 (0.39-1.22)
> Cut-off=0.15(0-1.17)
< Cut-off=0.15(0-1.17)
P = 0.037
HR (95%CI) = 4.36 (2.06-10.7)
> Cut-off=0.15(0.01-0.66)
< Cut-off=0.15(0.01-0.66)
P = 0.044
HR (95%CI) = 3.58 (1.52-8.44)
Survival probability
Survival time (days)
Number at risk
Group

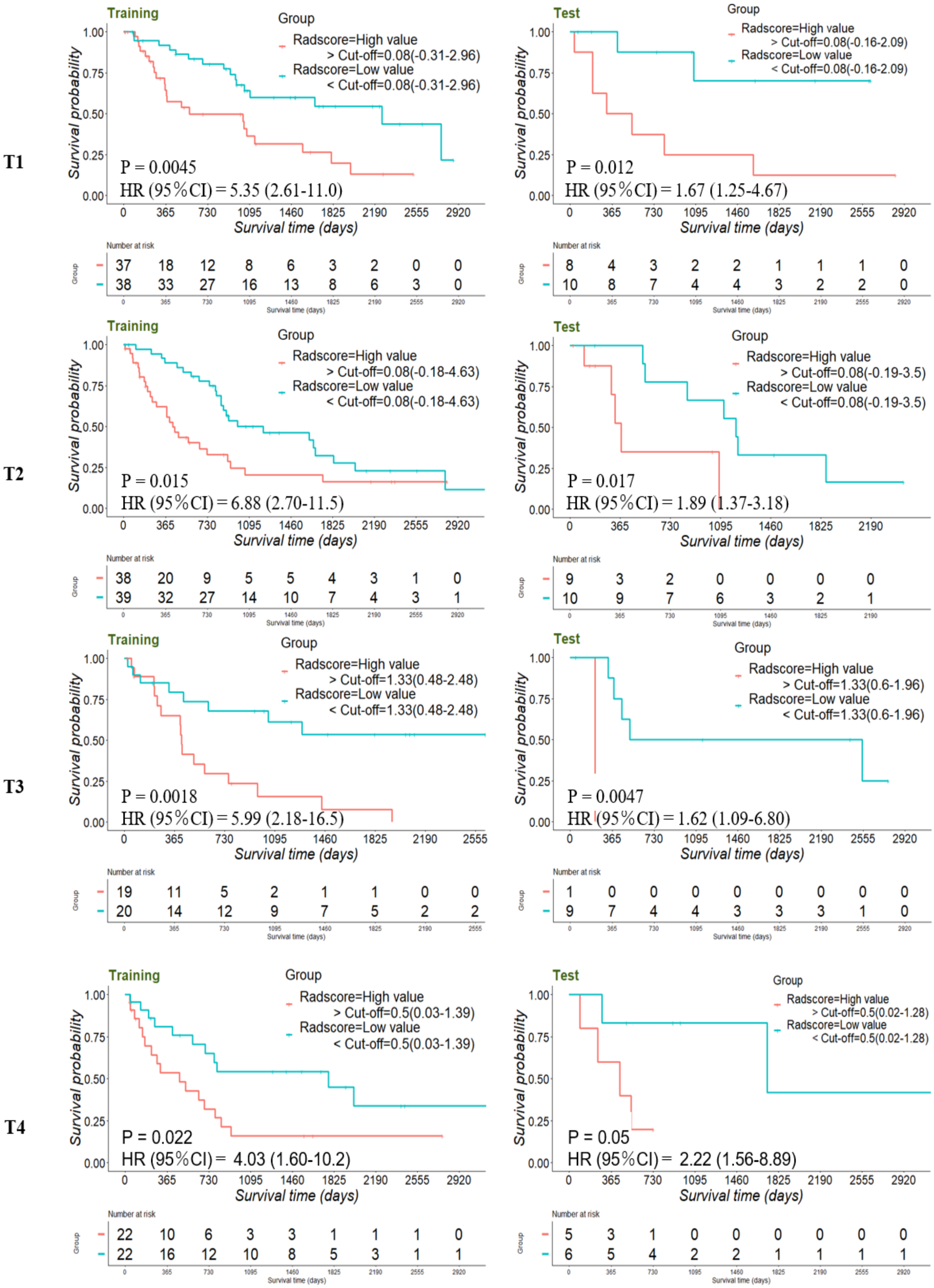
T1
Training
Group
Radscore=High value > Cut-off=0.08(-0.31-2.96)
Radscore=Low value < Cut-off=0.08(-0.31-2.96)
P = 0.0045
HR (95%CI) = 5.35 (2.61-11.0)
Test
Radscore=High value > Cut-off=0.08(-0.16-2.09)
Radscore=Low value < Cut-off=0.08(-0.16-2.09)
P = 0.012
HR (95%CI) = 1.67 (1.25-4.67)
T2
Training
Radscore=High value > Cut-off=0.08(-0.18-4.63)
Radscore=Low value < Cut-off=0.08(-0.18-4.63)
P = 0.015
HR (95%CI) = 6.88 (2.70-11.5)
Test
Radscore=High value > Cut-off=0.08(-0.19-3.5)
Radscore=Low value < Cut-off=0.08(-0.19-3.5)
P = 0.017
HR (95%CI) = 1.89 (1.37-3.18)
T3
Training
Radscore=High value > Cut-off=1.33(0.48-2.48)
Radscore=Low value < Cut-off=1.33(0.48-2.48)
P = 0.0018
HR (95%CI) = 5.99 (2.18-16.5)
Test
Radscore=High value > Cut-off=1.33(0.6-1.96)
Radscore=Low value < Cut-off=1.33(0.6-1.96)
P = 0.0047
HR (95%CI) = 1.62 (1.09-6.80)
T4
Training
Radscore=High value > Cut-off=0.5(0.03-1.39)
Radscore=Low value < Cut-off=0.5(0.03-1.39)
P = 0.022
HR (95%CI) = 4.03 (1.60-10.2)
Test
Radscore=High value > Cut-off=0.5(0.02-1.28)
Radscore=Low value < Cut-off=0.5(0.02-1.28)
P = 0.05
HR (95%CI) = 2.22 (1.56-8.89)
Survival probability
Survival time (days)
Number at risk

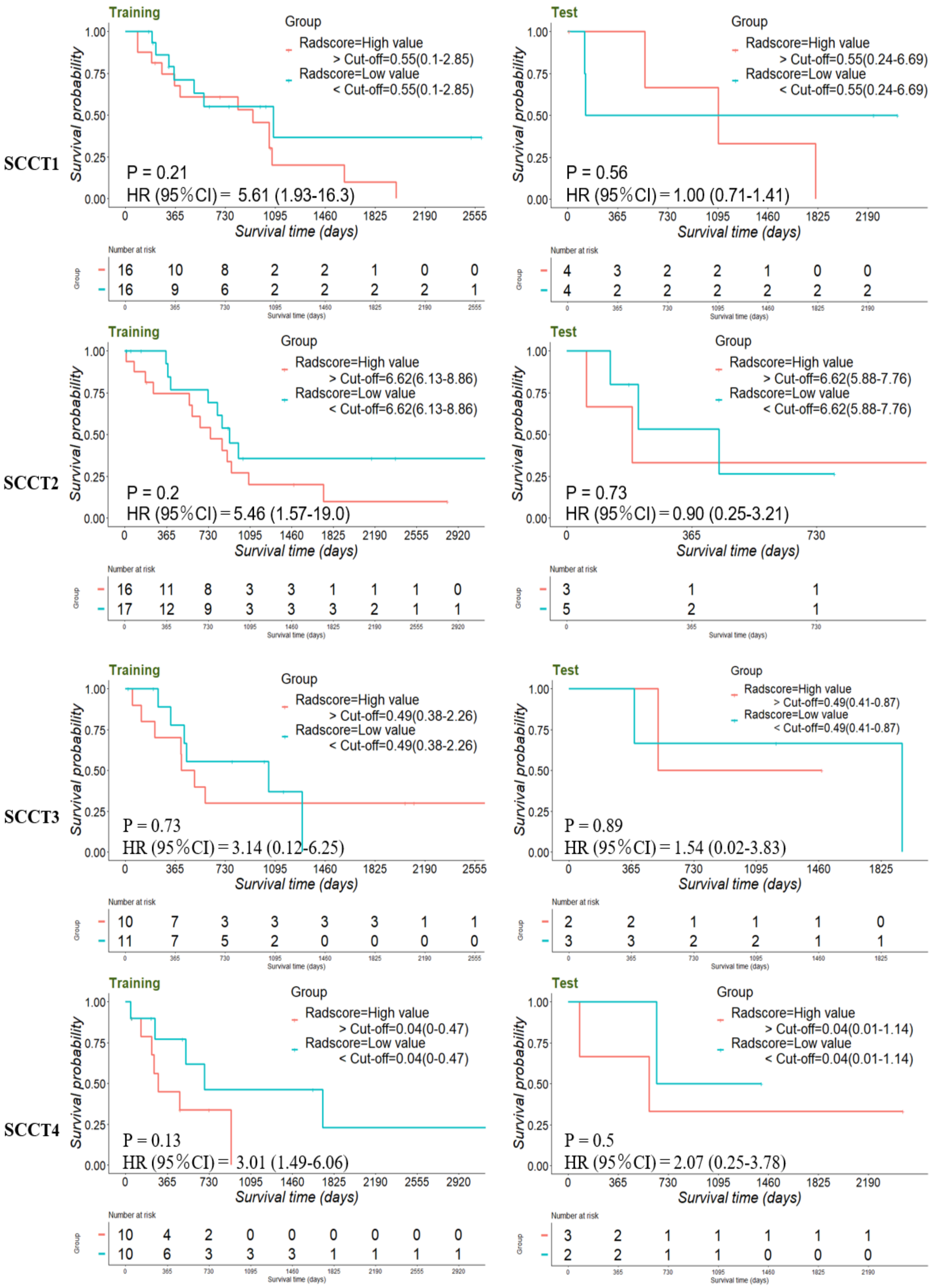
SCCT1
Training
Group
Radscore=High value > Cut-off=0.55(0.1-2.85)
Radscore=Low value < Cut-off=0.55(0.1-2.85)
P = 0.21
HR (95%CI) = 5.61 (1.93-16.3)
Survival probability
Survival time (days)
Number at risk
Test
Group
Radscore=High value > Cut-off=0.55(0.24-6.69)
Radscore=Low value < Cut-off=0.55(0.24-6.69)
P = 0.56
HR (95%CI) = 1.00 (0.71-1.41)
SCCT2
Training
Group
Radscore=High value > Cut-off=6.62(6.13-8.86)
Radscore=Low value < Cut-off=6.62(6.13-8.86)
P = 0.2
HR (95%CI) = 5.46 (1.57-19.0)
Test
Group
Radscore=High value > Cut-off=6.62(5.88-7.76)
Radscore=Low value < Cut-off=6.62(5.88-7.76)
P = 0.73
HR (95%CI) = 0.90 (0.25-3.21)
SCCT3
Training
Group
Radscore=High value > Cut-off=0.49(0.38-2.26)
Radscore=Low value < Cut-off=0.49(0.38-2.26)
P = 0.73
HR (95%CI) = 3.14 (0.12-6.25)
Test
Group
Radscore=High value > Cut-off=0.49(0.41-0.87)
Radscore=Low value < Cut-off=0.49(0.41-0.87)
P = 0.89
HR (95%CI) = 1.54 (0.02-3.83)
SCCT4
Training
Group
Radscore=High value > Cut-off=0.04(0-0.47)
Radscore=Low value < Cut-off=0.04(0-0.47)
P = 0.13
HR (95%CI) = 3.01 (1.49-6.06)
Test
Group
Radscore=High value > Cut-off=0.04(0.01-1.14)
Radscore=Low value < Cut-off=0.04(0.01-1.14)
P = 0.5
HR (95%CI) = 2.07 (0.25-3.78)

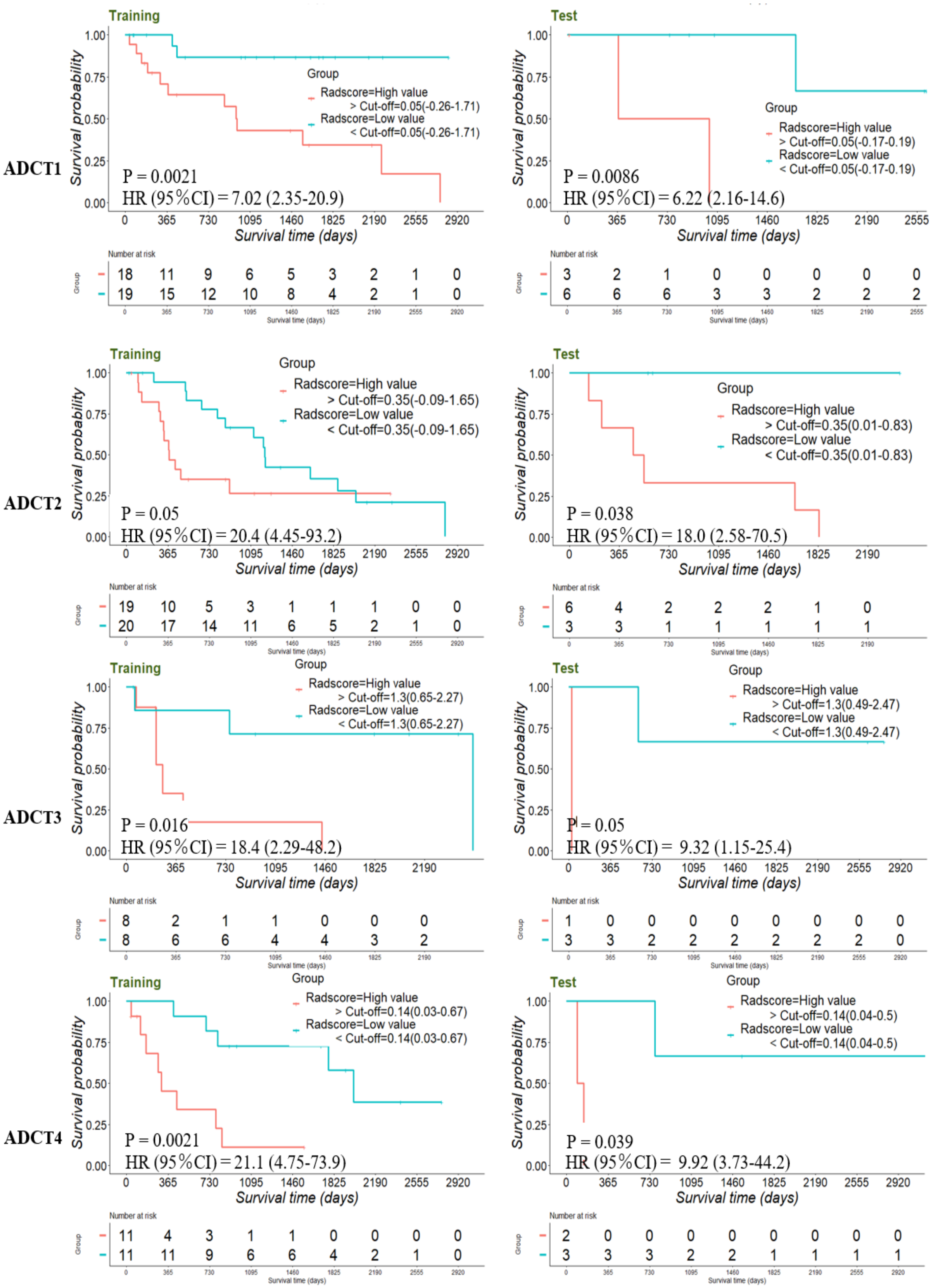

Abbreviation: HR = Hazard ratio, CI = confidence interval

Supplementary I: Kaplan–Meier curves for low- and high-risk groups based on the rad score in the radiomic models for each subgroup. To avoid complications, the case when it was closest to the mean C-index of the test dataset among the five-fold cross-validation is shown.

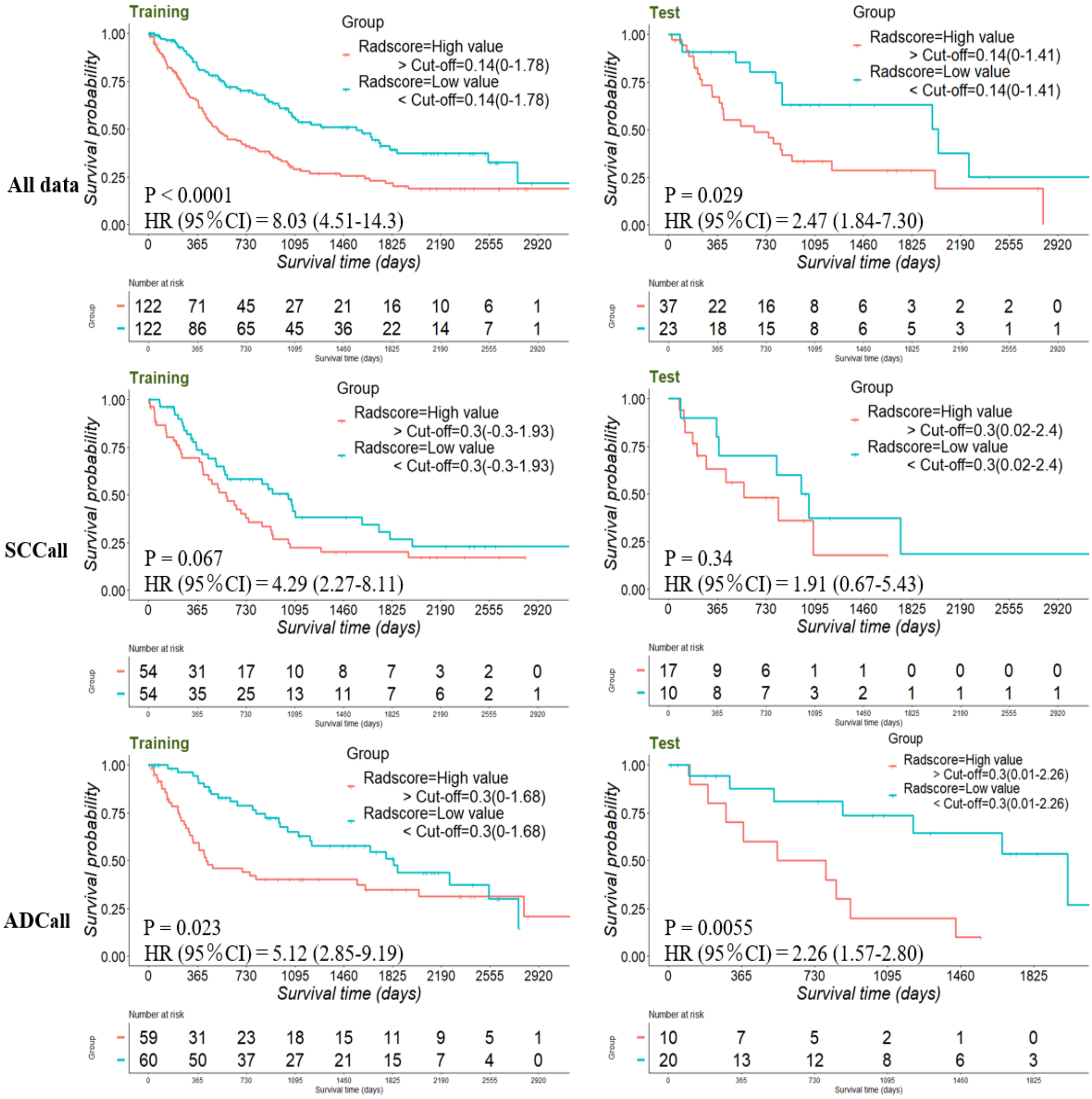
All data
SCCall
ADCall
Training
Test
Group
Radscore=High value
> Cut-off=0.14(0-1.78)
Radscore=Low value
< Cut-off=0.14(0-1.78)
P < 0.0001
HR (95%CI) = 8.03 (4.51-14.3)
> Cut-off=0.14(0-1.41)
< Cut-off=0.14(0-1.41)
P = 0.029
HR (95%CI) = 2.47 (1.84-7.30)
> Cut-off=0.3(-0.3-1.93)
< Cut-off=0.3(-0.3-1.93)
P = 0.067
HR (95%CI) = 4.29 (2.27-8.11)
> Cut-off=0.3(0.02-2.4)
< Cut-off=0.3(0.02-2.4)
P = 0.34
HR (95%CI) = 1.91 (0.67-5.43)
> Cut-off=0.3(0-1.68)
< Cut-off=0.3(0-1.68)
P = 0.023
HR (95%CI) = 5.12 (2.85-9.19)
> Cut-off=0.3(0.01-2.26)
< Cut-off=0.3(0.01-2.26)
P = 0.0055
HR (95%CI) = 2.26 (1.57-2.80)
Survival probability
Survival time (days)
Number at risk
Group
122 71 45 27 21 16 10 6 1
122 86 65 45 36 22 14 7 1
37 22 16 8 6 3 2 2 0
23 18 15 8 6 5 3 1 1
54 31 17 10 8 7 3 2 0
54 35 25 13 11 7 6 2 1
17 9 6 1 1 0 0 0 0
10 8 7 3 2 1 1 1 1
59 31 23 18 15 11 9 5 1
60 50 37 27 21 15 7 4 0
10 7 5 2 1 0
20 13 12 8 6 3

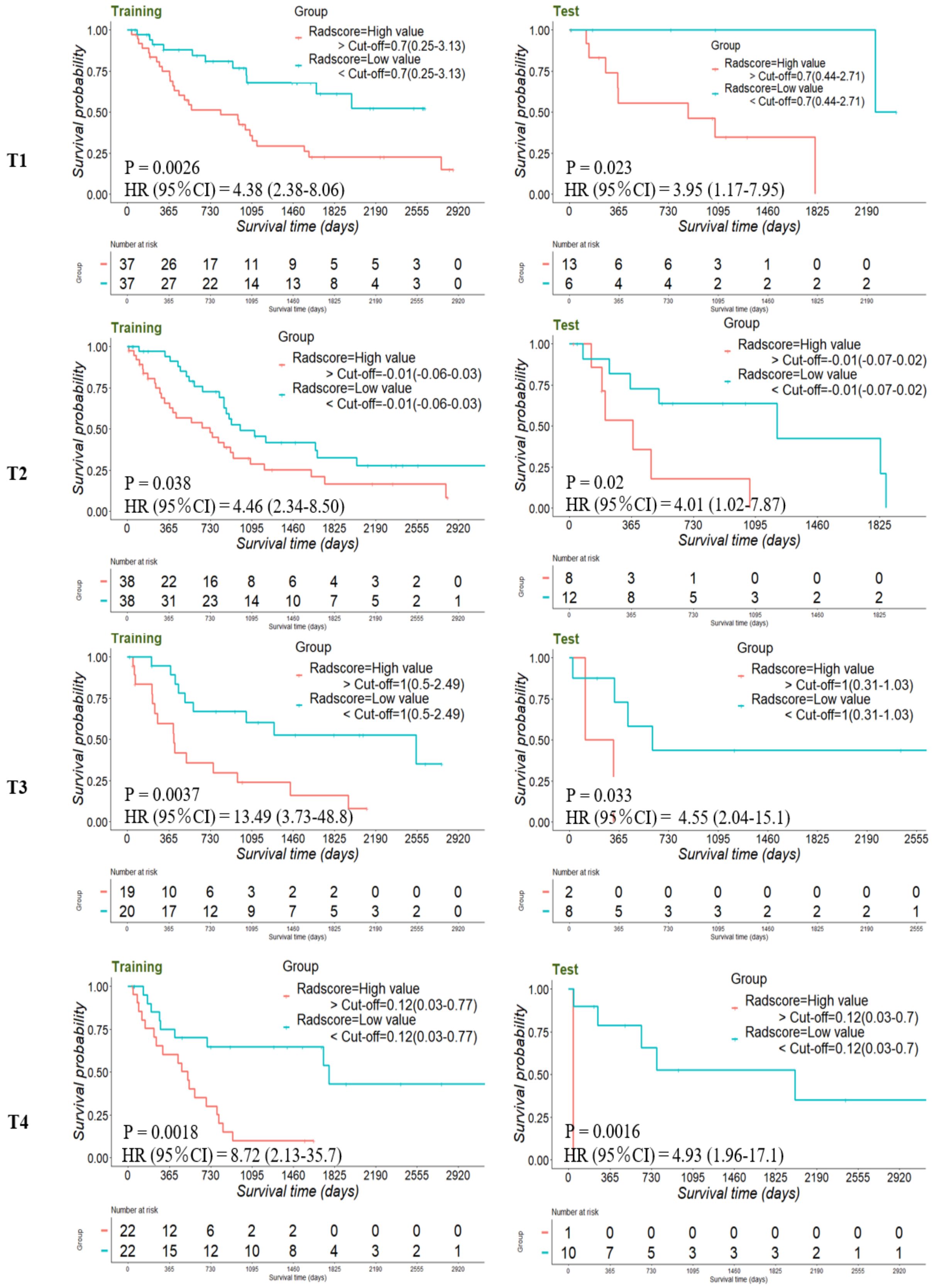
T1
Training
Group
Radscore=High value > Cut-off=0.7(0.25-3.13)
Radscore=Low value < Cut-off=0.7(0.25-3.13)
P = 0.0026
HR (95%CI) = 4.38 (2.38-8.06)
Survival probability
Survival time (days)
Number at risk
37 26 17 11 9 5 5 3 0
37 27 22 14 13 8 4 3 0
Test
Group
Radscore=High value > Cut-off=0.7(0.44-2.71)
Radscore=Low value < Cut-off=0.7(0.44-2.71)
P = 0.023
HR (95%CI) = 3.95 (1.17-7.95)
Number at risk
13 6 6 3 1 0 0
6 4 4 2 2 2 2
T2
Training
Group
Radscore=High value > Cut-off=-0.01(-0.06-0.03)
Radscore=Low value < Cut-off=-0.01(-0.06-0.03)
P = 0.038
HR (95%CI) = 4.46 (2.34-8.50)
Number at risk
38 22 16 8 6 4 3 2 0
38 31 23 14 10 7 5 2 1
Test
Group
Radscore=High value > Cut-off=-0.01(-0.07-0.02)
Radscore=Low value < Cut-off=-0.01(-0.07-0.02)
P = 0.02
HR (95%CI) = 4.01 (1.02-7.87)
Number at risk
8 3 1 0 0 0
12 8 5 3 2 2
T3
Training
Group
Radscore=High value > Cut-off=1(0.5-2.49)
Radscore=Low value < Cut-off=1(0.5-2.49)
P = 0.0037
HR (95%CI) = 13.49 (3.73-48.8)
Number at risk
19 10 6 3 2 2 0 0 0
20 17 12 9 7 5 3 2 0
Test
Group
Radscore=High value > Cut-off=1(0.31-1.03)
Radscore=Low value < Cut-off=1(0.31-1.03)
P = 0.033
HR (95%CI) = 4.55 (2.04-15.1)
Number at risk
2 0 0 0 0 0 0 0
8 5 3 3 2 2 2 1
T4
Training
Group
Radscore=High value > Cut-off=0.12(0.03-0.77)
Radscore=Low value < Cut-off=0.12(0.03-0.77)
P = 0.0018
HR (95%CI) = 8.72 (2.13-35.7)
Number at risk
22 12 6 2 2 0 0 0 0
22 15 12 10 8 4 3 2 1
Test
Group
Radscore=High value > Cut-off=0.12(0.03-0.7)
Radscore=Low value < Cut-off=0.12(0.03-0.7)
P = 0.0016
HR (95%CI) = 4.93 (1.96-17.1)
Number at risk
1 0 0 0 0 0 0 0 0
10 7 5 3 3 3 2 1 1

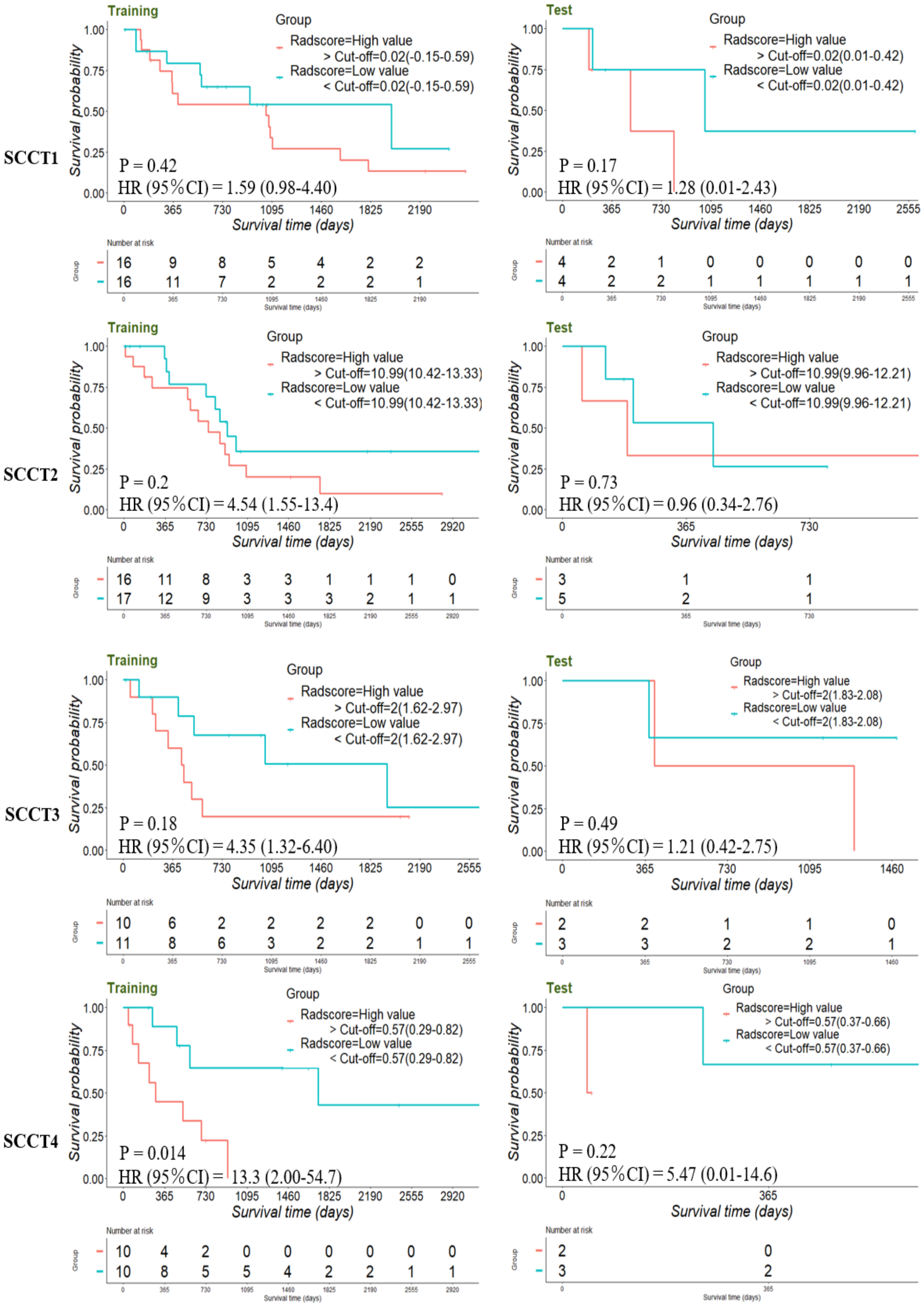

SCCT1
Training
Group
Radscore=High value
> Cut-off=0.02(-0.15-0.59)
Radscore=Low value
< Cut-off=0.02(-0.15-0.59)
P = 0.42
HR (95%CI) = 1.59 (0.98-4.40)
Test
Group
Radscore=High value
> Cut-off=0.02(0.01-0.42)
Radscore=Low value
< Cut-off=0.02(0.01-0.42)
P = 0.17
HR (95%CI) = 1.28 (0.01-2.43)
SCCT2
Training
Group
Radscore=High value
> Cut-off=10.99(10.42-13.33)
Radscore=Low value
< Cut-off=10.99(10.42-13.33)
P = 0.2
HR (95%CI) = 4.54 (1.55-13.4)
Test
Group
Radscore=High value
> Cut-off=10.99(9.96-12.21)
Radscore=Low value
< Cut-off=10.99(9.96-12.21)
P = 0.73
HR (95%CI) = 0.96 (0.34-2.76)
SCCT3
Training
Group
Radscore=High value
> Cut-off=2(1.62-2.97)
Radscore=Low value
< Cut-off=2(1.62-2.97)
P = 0.18
HR (95%CI) = 4.35 (1.32-6.40)
Test
Group
Radscore=High value
> Cut-off=2(1.83-2.08)
Radscore=Low value
< Cut-off=2(1.83-2.08)
P = 0.49
HR (95%CI) = 1.21 (0.42-2.75)
SCCT4
Training
Group
Radscore=High value
> Cut-off=0.57(0.29-0.82)
Radscore=Low value
< Cut-off=0.57(0.29-0.82)
P = 0.014
HR (95%CI) = 13.3 (2.00-54.7)
Test
Group
Radscore=High value
> Cut-off=0.57(0.37-0.66)
Radscore=Low value
< Cut-off=0.57(0.37-0.66)
P = 0.22
HR (95%CI) = 5.47 (0.01-14.6)
Survival probability
Survival time (days)
Number at risk

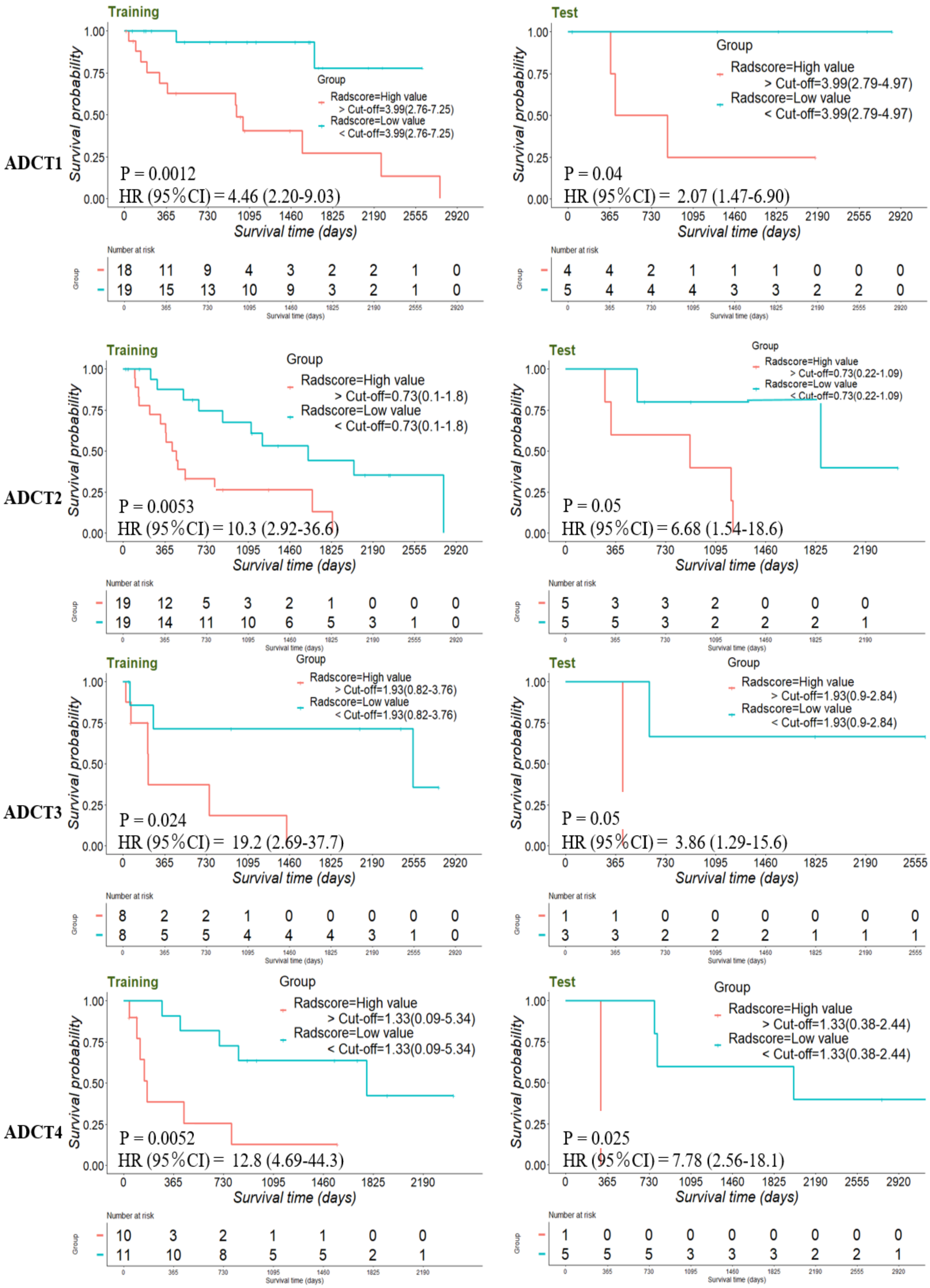

Abbreviation: HR = Hazard ratio, CI = confidence interval

Supplementary J: Kaplan-Meier curves for low- and high-risk groups based on the rad score in the combined models for each subgroup. To avoid complications, the case when it was closest to the mean C-index of the test dataset among the five-fold cross-validation is shown.

Supplementary K: For each analysis group, the features and their coefficients selected by the LASSO Cox regression model in the radiomic models.

Subgroup	fold	Features (Coefficient)
All data	1	first-order_10Percentile (4.08×10^{-4}), first-order_Kurtosis (4.69×10^{-4}), GLCM_ClusterProminence (1.03×10^{-7}), GLSZM_LargeAreaEmphasis (1.71×10^{-7}), GLSZM_SmallAreaEmphasis (7.62×10^{-1})
	2	first-order_10Percentile (8.99×10^{-6}), first-order_Kurtosis (3.43×10^{-3}), first-order_Maximum (4.58×10^{-5}), GLSZM_LargeAreaEmphasis (2.22×10^{-8})
	3	Shape_MajorAxisLength (4.16×10^{-4}), first-order_90Percentile (1.27×10^{-4}), GLSZM_LargeAreaEmphasis (1.22×10^{-7})
	4	Shape_MajorAxisLength (2.49×10^{-4}), first-order_90Percentile (4.27×10^{-4}), GLSZM_LargeAreaEmphasis (1.02×10^{-7})
	5	Shape_MajorAxisLength (2.31×10^{-3}), first-order_90Percentile (2.02×10^{-4}), first-order_Maximum (7.98×10^{-6}), GLDM_DependenceVariance (1.52×10^{-3}), GLSZM_LargeAreaEmphasis (7.42×10^{-8})
SCC_{all}	1	first-order_10Percentile (3.44×10^{-4}), first-order_90Percentile (2.81×10^{-3}), first-order_Kurtosis (2.97×10^{-3}), GLCM_DependenceVariance (2.97), GLSZM_GrayLevelNonUniformityNormalized (2.33×10^{1}), GLSZM_SizeZoneNonUniformityNormalized (2.37)
	2	GLCM_SumAverage (7.36×10^{-3}), GLSZM_GrayLevelNonUniformityNormalized (7.73), GLSZM_LargeAreaEmphasis (2.76×10^{-7})
	3	Shape_Maximum2DDiameterColumn (2.13×10^{-3}), first-order_90Percentile (8.69×10^{-4}), first-order_Kurtosis (1.03×10^{-2})
	4	first-order_90Percentile (9.66×10^{-4}), GLSZM_GrayLevelVariance (5.82×10^{-3}) GLSZM_LargeAreaLowGrayLevelEmphasis (2.59×10^{-4})
	5	first-order_90Percentile (4.79×10^{-5}), GLCM_SumAverage (6.11×10^{-4})
ADC_{all}	1	first-order_Maximum (2.47×10^{-4}), GLSZM_GrayLevelVariance (1.62×10^{-3})
	2	Shape_Maximum2DDiameterRow (6.88×10^{-3}), first-order_Energy (1.18×10^{-11}), first-order_Maximum (6.18×10^{-5})
	3	Shape_MajorAxisLength (3.33×10^{-4}), first-order_Maximum (5.91×10^{-5}), GLSZM_LargeAreaHighGrayLevelEmphasis (2.03×10^{-11})
	4	Shape_MajorAxisLength (6.68×10^{-3}), first-order_10Percentile (2.78×10^{-4}), first-order_Maximum (6.71×10^{-5}), GLDM_LargeDependenceHighGrayLevelEmphasis (5.12×10^{-7}), GLSZM_LargeAreaEmphasis (2.67×10^{-8}), NGTDM_Busyness (5.42×10^{-2})
	5	first-order_Maximum (1.76×10^{-4}), GLRLM_LongRunHighGrayLevelEmphasis (3.59×10^{-5})
T1	1	first-order_90Percentile (1.41×10^{-4}), GLSZM_LargeAreaHighGrayLevelEmphasis (4.34×10^{-12})

	2	GLSZM_GrayLevelNonUniformity (3.89×10^{-3}), GLSZM_SizeZoneNonUniformityNormalized (2.45×10^{-1}), NGTDM_Strength (2.65×10^{-3})
	3	first-order_90Percentile (3.26×10^{-4}), first-order_Maximum (5.44×10^{-4}), GLSZM_GrayLevelNonUniformity (1.18×10^{-3})
	4	first-order_90Percentile (2.15×10^{-3}), first-order_Energy (2.12×10^{-11}), first-order_TotalEnergy (3.05×10^{-15}), GLSZM_LargeAreaHighGrayLevelEmphasis (1.29×10^{-9}), GLSZM_SmallAreaEmphasis (2.63×10^{-1})
	5	first-order_90Percentile (3.91×10^{-4}), GLSZM_GrayLevelNonUniformity (3.78×10^{-3}), GLSZM_LargeAreaHighGrayLevelEmphasis (1.41×10^{-9}), GLSZM_SmallAreaEmphasis (9.07×10^{-1})
T2	1	first-order_10Percentile (6.47×10^{-4}), GLSZM_SmallAreaLowGrayLevelEmphasis (8.76×10^{1})
	2	first-order_Kurtosis (1.48×10^{-2}), first-order_Maximum (4.51×10^{-4})
	3	Shape_MajorAxisLength (2.38×10^{-3}), GLCM_Idmn (7.54), GLSZM_LargeAreaEmphasis (1.34×10^{-7}), GLSZM_SmallAreaLowGrayLevelEmphasis (7.48×10^{1})
	4	first-order_10Percentile (5.55×10^{-4}), first-order_Energy (5.01×10^{-11}), first-order_TotalEnergy (6.45×10^{-12}), GLDM_LargeDependenceLowGrayLevelEmphasis (3.25×10^{-1}), GLSZM_LargeAreaEmphasis (9.94×10^{-8}), GLSZM_SmallAreaLowGrayLevelEmphasis (1.92×10^{1})
	5	first-order_10Percentile (1.80×10^{-4}), first-order_Maximum (2.73×10^{-6})
T3	1	GLRLM_LongRunLowGrayLevelEmphasis (3.64×10^{1}), GLSZM_HighGrayLevelZoneEmphasis (1.72×10^{-3})
	2	Shape_MajorAxisLength (6.08×10^{-3}), GLSZM_LargeAreaLowGrayLevelEmphasis (1.84×10^{-4})
	3	GLSZM_GrayLevelNonUniformityNormalized (1.03×10^{1}), GLSZM_GrayLevelVariance (3.58×10^{-3}), GLSZM_LargeAreaLowGrayLevelEmphasis (8.42×10^{-5})
	4	first-order_Kurtosis (9.61×10^{-3}), GLSZM_GrayLevelVariance (4.64×10^{-5})
	5	Shape_MajorAxisLength (8.69×10^{-4}), GLSZM_LargeAreaLowGrayLevelEmphasis (6.26×10^{-5})
T4	1	Shape_MajorAxisLength (1.85×10^{-2}), first-order_Mean (2.41×10^{-3}), GLSZM_HighGrayLevelZoneEmphasis (2.06×10^{-4}), GLSZM_SmallAreaEmphasis (1.75)
	2	GLSZM_HighGrayLevelZoneEmphasis (5.16×10^{-4}), GLSZM_LargeAreaEmphasis (6.88×10^{-8})
	3	Shape_Maximum2DDiameterColumn (1.45×10^{-3}), GLRLM_LongRunHighGrayLevelEmphasis (1.14×10^{-5}), GLSZM_HighGrayLevelZoneEmphasis (1.46×10^{-5})
	4	Shape_Maximum2DDiameterColumn (3.98×10^{-3}), first-order_Mean (2.10×10^{-4}), first-order_Skewness (1.08×10^{-2}), GLDM_LargeDependenceHighGrayLevelEmphasis (4.37×10^{-7}), GLSZM_HighGrayLevelZoneEmphasis (6.40×10^{-4}), GLSZM_SmallAreaEmphasis (2.41)
	5	Shape_MajorAxisLength (1.28×10^{-2}), first-order_Mean (6.51×10^{-4})
SCC$_{T1}$	1	Shape_Sphericity (4.45), Shape_SurfaceVolumeRatio (5.67), first-order_90Percentile (2.78×10^{-3}), first-order_Energy (2.42×10^{-10}), first-order_TotalEnergy (1.40×10^{-14})
	2	Shape_Maximum2DDiameterColumn (6.93×10^{-4}), GLCM_Correlation (2.59×10^{-1})

	3	first-order_90Percentile (1.65×10^{-3}), first-order_Energy (1.29×10^{-10}), first-order_TotalEnergy (8.12×10^{-26})
	4	first-order_90Percentile (2.48×10^{-3}), GLDM_LargeDependenceLowGrayLevelEmphasis (1.25×10^{-2})
	5	first-order_Energy (8.08×10^{-11}), first-order_TotalEnergy (3.49×10^{-26})
SCC$_{T2}$	1	first-order_10Percentile (2.47×10^{-4}), GLSZM_SmallAreaLowGrayLevelEmphasis (5.19×10^{1})
	2	first-order_10Percentile (3.28×10^{-4}), GLCM_Imc1 (3.48×10^{-1})
	3	first-order_10Percentile (1.54×10^{-4}), GLCM_InverseVariance (2.11), GLSZM_SizeZoneNonUniformityNormalized (6.16), GLSZM_SmallAreaLowGrayLevelEmphasis (6.61)
	4	first-order_10Percentile (8.96×10^{-4}), first-order_Maximum (9.13×10^{-4}) , GLCM_Imc1 (2.53×10^{-1})
	5	Shape_SurfaceVolumeRatio (2.90×10^{-1}), first-order_10Percentile (1.15×10^{-4}), first-order_Maximum (2.32×10^{-3}), GLCM_Imc1 (5.14), GLSZM_SmallAreaLowGrayLevelEmphasis (7.56×10^{1})
SCC$_{T3}$	1	first-order_10Percentile (2.52×10^{-4}), GLCM_Correlation (9.70×10^{-1}), GLSZM_LargeAreaLowGrayLevelEmphasis (8.44×10^{-4})
	2	GLDM_DependenceVariance (6.43×10^{-2}), GLRLM_LongRunLowGrayLevelEmphasis (1.82×10^{1}), GLSZM_LargeAreaLowGrayLevelEmphasis (6.04×10^{-4}), GLSZM_ZoneEntropy (1.93)
	3	GLCM_Correlation (5.67), GLSZM_LargeAreaLowGrayLevelEmphasis (3.25×10^{-4})
	4	GLCM_Correlation (6.22×10^{-1}), GLSZM_LargeAreaLowGrayLevelEmphasis (2.84×10^{-4})
	5	GLCM_Correlation (6.37), GLSZM_GrayLevelNonUniformityNormalized (3.46)
SCC$_{T4}$	1	first-order_Energy (1.49×10^{-11}), first-order_TotalEnergy (2.01×10^{-12})
	2	Shape_MajorAxisLength (3.59×10^{-3}), first-order_Energy (2.09×10^{-11}), first-order_TotalEnergy (9.91×10^{-12})
	3	first-order_Energy (7.96×10^{-11}), first-order_TotalEnergy (8.39×10^{-14})
	4	Shape_MajorAxisLength (5.10×10^{-3}), GLDM_DependenceNonUniformityNormalized (6.98)
	5	first-order_Energy (5.98×10^{-11}), first-order_TotalEnergy (1.60×10^{-11})
ADC$_{T1}$	1	first-order_Maximum (1.87×10^{-3}), GLCM_Imc1 (1.82)
	2	Shape_Flatness (1.41), first-order_Maximum (2.23×10^{-3}), GLCM_Imc1 (5.14)
	3	Shape_Flatness (2.98), first-order_Maximum (3.81×10^{-12}), first-order_Energy (1.82×10^{-3}), GLCM_Imc1 (3.09), GLSZM_LargeAreaEmphasis (1.59×10^{-6})
	4	Shape_Flatness (8.37×10^{-1}), first-order_Maximum (9.17×10^{-4}), GLCM_Imc1 (5.79), GLDM_DependenceNonUniformity (9.15×10^{-5})
	5	Shape_Flatness (1.13), first-order_Energy (2.59×10^{-12}), first-order_Maximum (2.15×10^{-3}), GLCM_Imc1 (1.28×10^{1}), GLSZM_LargeAreaEmphasis (3.11×10^{-7})
ADC$_{T2}$	1	Shape_Elongation (3.77×10^{-1}), first-order_Energy (3.21×10^{-10}), GLDM_DependenceVariance (1.14×10^{-2}), GLSZM_GrayLevelNonUniformityNormalized (1.12×10^{1})
	2	first-order_Energy (1.01×10^{-10}), first-order_Maximum (2.49×10^{-4}), GLRLM_GrayLevelNonUniformityNormalized (2.95),

		GLSZM_GrayLevelNonUniformityNormalized (7.29)
	3	first-order_Maximum (5.36×10^{-4}), NGTDM_Busyness (8.97×10^{-3})
	4	first-order_Energy (6.01×10^{-11}), first-order_TotalEnergy (4.04×10^{-25})
	5	first-order_Energy (4.33×10^{-11}), first-order_Maximum (1.55×10^{-4}), first-order_TotalEnergy (6.23×10^{-13})
ADC_{T3}	1	Shape_MajorAxisLength (3.77×10^{-1}), first-order_Kurtosis (2.52×10^{-4}), GLSZM_HighGrayLevelZoneEmphasis (8.44×10^{-4})
	2	Shape_MajorAxisLength (3.77×10^{-1}), first-order_Kurtosis (2.52×10^{-4}), GLCM_InverseVariance (6.43×10^{-2})
	3	Shape_MajorAxisLength (3.77×10^{-1}), first-order_Kurtosis (2.52×10^{-4}), GLCM_ClusterShade (5.67)
	4	Shape_MajorAxisLength (3.77×10^{-1}), GLRLM_GrayLevelNonUniformityNormalized (2.95)
	5	first-order_Kurtosis (2.52×10^{-4}), GLSZM_HighGrayLevelZoneEmphasis (3.46)
ADC_{T4}	1	Shape_Maximum2DDiameterRow (7.74×10^{-3}), GLRLM_LongRunHighGrayLevelEmphasis (1.28×10^{-6})
	2	Shape_MajorAxisLength (1.28×10^{-2}), first-order_10Percentile (9.16×10^{-4})
	3	Shape_Maximum2DDiameterRow (6.22×10^{-3}), first-order_Mean (2.02×10^{-3}), GLCM_ClusterShade (4.94×10^{-5}), GLSZM_HighGrayLevelZoneEmphasis (5.35×10^{-4})
	4	first-order_10Percentile (3.64×10^{-5}), first-order_Mean (3.64×10^{-3}), GLCM_ClusterProminence (6.95×10^{-7}), GLRLM_LongRunHighGrayLevelEmphasis (1.39×10^{-4}), GLSZM_LargeAreaHighGrayLevelEmphasis (2.09×10^{-10})
	5	Shape_Maximum2DDiameterRow (5.22×10^{-3}), first-order_10Percentile (2.32×10^{-3}), GLCM_ClusterProminence (1.72×10^{-6})

Abbreviation: SCC = squamous cell carcinoma, ADC = adenocarcinoma,

GLCM = gray-level co-occurrence matrix, GLDM = gray-level dependence matrix,

GLRLM = gray-level run length matrix, GLSZM = gray-level size zone matrix,

NGTDM = neighborhood gray tone difference matrix

Supplementary L: For each analysis group, the features and their coefficients selected by the LASSO Cox regression model in the combined models.

Subgroup	fold	Features (Coefficient)
All data	1	Shape_LargeAreaEmphasis (1.47×10^{-7}), N stage (6.82×10^{-2}), M stage (1.23×10^{-1})
	2	first-order_Maximum (7.06×10^{-7}), GLSZM_LargeAreaEmphasis (8.16×10^{-8}), N stage (6.60×10^{-2}), M stage (3.25×10^{-1})
	3	first-order_10Percentile (5.62×10^{-4}), first-order_90Percentile (1.40×10^{-3}), first-order_Kurtosis (8.79×10^{-4}), GLCM_Correlation (3.71×10^{-1}), GLSZM_LargeAreaEmphasis (4.58×10^{-8}), Age (4.65×10^{-3}), N stage (1.70×10^{-1}), M stage (3.79×10^{-1}), Clinical stage (1.22×10^{-2})
	4	first-order_Kurtosis (1.83×10^{-4}), GLSZM_LargeAreaEmphasis (3.74×10^{-8}), N stage (5.89×10^{-2}), M stage (2.96×10^{-2})
	5	Shape_MajorAxisLength (3.04×10^{-5}), first-order_90Percentile (1.43×10^{-4}), N stage (9.09×10^{-2}), M stage (5.51×10^{-1})
SCC_{all}	1	first-order_90Percentile (6.34×10^{-4}), first-order_Kurtosis (1.99×10^{-3}), GLSZM_LargeAreaEmphasis (2.41×10^{-7}), N stage (1.94×10^{-1}), Clinical stage (4.90×10^{-3})
	2	first-order_90Percentile (9.62×10^{-4}), GLSZM_GrayLevelVariance (7.20×10^{-3}), GLSZM_LargeAreaLowGrayLevelEmphasis (2.81×10^{-4}), Age (2.02×10^{-2}), N stage (3.45×10^{-2}), Clinical stage (6.67×10^{-2})
	3	first-order_90Percentile (2.16×10^{-4}), M stage (5.30×10^{-2})
	4	first-order_90Percentile (9.41×10^{-4}), GLSZM_LargeAreaEmphasis (2.91×10^{-8}), N stage (1.39×10^{-1})
	5	GLSZM_LargeAreaEmphasis (2.15×10^{-8}), N stage (1.43×10^{-1}), M stage (6.69×10^{-1})
ADC_{all}	1	first-order_Maximum (1.08×10^{-4}), GLRLM_LongRunHighGrayLevelEmphasis (2.83×10^{-5}), GLSZM_SmallAreaEmphasis (1.01), NGTDM_Complexity (1.26×10^{-5}), N stage (8.10×10^{-2}), M stage (4.85×10^{-1}), Clinical stage (8.26×10^{-2})
	2	first-order_Maximum (1.34×10^{-4}), N stage (1.12×10^{-1}), M stage (4.91×10^{-1})
	3	first-order_Maximum (8.41×10^{-5}), GLDM_LargeDependenceHighGrayLevelEmphasis (3.86×10^{-7}), N stage (2.19×10^{-1}), M stage (7.86×10^{-1})
	4	first-order_10Percentile (1.20×10^{-4}), first-order_Maximum (1.07×10^{-4}), N stage (2.49×10^{-1}), M stage (2.74×10^{-1})
	5	Shape_MajorAxisLength (3.67×10^{-4}), first-order_Maximum (4.88×10^{-5}), GLRLM_LongRunHighGrayLevelEmphasis (1.94×10^{-5}), N stage (1.01×10^{-1}), M stage (2.59×10^{-1})
T1	1	GLSZM_GrayLevelNonUniformity (2.75×10^{-3}), GLSZM_LargeAreaHighGrayLevelEmphasis (9.35×10^{-10}), Clinical stage (1.98×10^{-1})
	2	first-order_90Percentile (9.94×10^{-5}), first-order_Maximum (2.84×10^{-4}), GLSZM_GrayLevelNonUniformity (4.38×10^{-3}),

		GLSZM_LargeAreaLowGrayLevelEmphasis (9.31×10^{-5}), NGTDM_Strength (2.67×10^{-2}), M stage (1.91), Clinical stage (2.78×10^{-1})
	3	GLCM_SumAverage (5.78×10^{-3}), GLSZM_GrayLevelNonUniformity (2.73×10^{-3}), Clinical stage (1.86×10^{-2})
	4	GLSZM_GrayLevelNonUniformity (2.93×10^{-3}), Clinical stage (6.98×10^{-3}), Histological subtype (8.63×10^{-2})
	5	Shape_Sphericity (3.62×10^{-1}), first-order_90Percentile (2.48×10^{-3}), first-order_Energy (3.09×10^{-11}), first-order_TotalEnergy (1.69×10^{-25}), GLSZM_LargeAreaHighGrayLevelEmphasis (1.14×10^{-9}), GLSZM_LargeAreaLowGrayLevelEmphasis (1.56×10^{-5}), GLSZM_SmallAreaEmphasis (1.18), N stage (6.62×10^{-2}), Clinical stage (2.53×10^{-1})
T2	1	first-order_10Percentile (1.49×10^{-4}), N stage (1.21×10^{-2})
	2	first-order_10Percentile (1.65×10^{-4}), GLCM_InverseVariance (1.05), N stage (7.81×10^{-3})
	3	first-order_10Percentile (8.58×10^{-5}), N stage (4.07×10^{-2})
	4	first-order_Energy (5.49×10^{-11}), first-order_Kurtosis (2.04×10^{-2}), first-order_TotalEnergy (9.20×10^{-12}), GLSZM_LargeAreaEmphasis (1.30×10^{-7}), GLSZM_SizeZoneNonUniformityNormalized (3.58×10^{-2}), GLSZM_SmallAreaLowGrayLevelEmphasis (1.59×10^{2}), Age (3.82×10^{-2}), N stage (4.25×10^{-1}), Histological subtype (1.22×10^{-2})
	5	first-order_Maximum (2.60×10^{-4}), N stage (4.30×10^{-2})
T3	1	Shape_MajorAxisLength (1.85×10^{-2}), first-order_Mean (2.41×10^{-3}), GLSZM_HighGrayLevelZoneEmphasis (2.06×10^{-4}), GLSZM_SmallAreaEmphasis (1.75), M stage (8.35×10^{-1})
	2	GLSZM_HighGrayLevelZoneEmphasis (5.16×10^{-4}), GLSZM_LargeAreaEmphasis (6.88×10^{-8}), M stage (8.69×10^{-2}), Clinical stage (6.90×10^{-1})
	3	Shape_Maximum2DDiameterColumn (1.45×10^{-3}), GLRLM_LongRunHighGrayLevelEmphasis (1.14×10^{-5}), GLSZM_HighGrayLevelZoneEmphasis (1.46×10^{-5}), M stage (4.22×10^{-2})
	4	Shape_Maximum2DDiameterColumn (3.98×10^{-3}), first-order_Mean (2.10×10^{-4}), first-order_Skewness (1.08×10^{-2}), GLDM_LargeDependenceHighGrayLevelEmphasis (4.37×10^{-7}), GLSZM_HighGrayLevelZoneEmphasis (6.40×10^{-4}), GLSZM_SmallAreaEmphasis (2.41), Clinical stage (3.90×10^{-2})
	5	Shape_MajorAxisLength (1.28×10^{-2}), first-order_Mean (6.51×10^{-1}), M stage (8.76×10^{-2}), Clinical stage (5.36×10^{-1})
T4	1	Shape_MajorAxisLength (8.77×10^{-3}), M stage (8.84×10^{-1}), Clinical stage (1.00×10^{-1}), Histological subtype (1.61×10^{-1})
	2	Shape_Maximum2DDiameterColumn (1.65×10^{-4}), GLDM_LargeDependenceHighGrayLevelEmphasis (9.03×10^{-8}),

		M stage (6.50×10^{-1}), Clinical stage (7.96×10^{-15})
	3	Shape_Maximum2DDiameterColumn (1.18×10^{-3}), M stage (7.38×10^{-1}), Clinical stage (7.76×10^{-15})
	4	Shape_Maximum2DDiameterColumn (4.44×10^{-4}), GLRLM_LongRunHighGrayLevelEmphasis (2.14×10^{-5}), GLSZM_GrayLevelVariance (2.86×10^{-3}), M stage (5.75×10^{-1}), Clinical stage (1.35×10^{-14})
	5	Shape_Maximum2DDiameterColumn (3.59×10^{-3}), first-order_Mean (1.93×10^{-3}), GLSZM_GrayLevelVariance (1.01×10^{-3}), GLSZM_HighGrayLevelZoneEmphasis (2.99×10^{-4}), GLSZM_ZoneEntropy (1.21×10^{-1}), M stage (3.32×10^{-1}), Clinical stage (4.11×10^{-16})
SCC_{T1}	1	GLCM_Correlation (2.61×10^{-3}), N stage (1.40×10^{-1})
	2	first-order_90Percentile (3.07×10^{-5}), GLCM_Correlation (5.97×10^{-1}), N stage (2.98×10^{-1})
	3	first-order_90Percentile (1.39×10^{-5}), GLCM_Correlation (2.59×10^{-2}), Clinical stage (1.53×10^{-1})
	4	first-order_90Percentile (9.62×10^{-4}), GLDM_LargeDependenceLowGrayLevelEmphasis (2.60×10^{-2}), N stage (5.49×10^{-3}), Clinical stage (5.55×10^{-3})
	5	first-order_Energy (1.38×10^{-10}), first-order_TotalEnergy (4.31×10^{-25}), GLDM_LargeDependenceLowGrayLevelEmphasis (1.99×10^{-2}), N stage (8.07×10^{-2})
SCC_{T2}	1	GLRLM_GrayLevelNonUniformityNormalized (6.53×10^{-1}), Age (5.69×10^{-2}), N stage (8.98×10^{-2})
	2	Shape_SurfaceVolumeRatio (1.63), first-order_10Percentile (4.73×10^{-4}), GLCM_Idmn (2.12), GLCM_Imc1 (1.34), Age (1.34×10^{-2}), M stage (1.43)
	3	Shape_SurfaceVolumeRatio (1.09×10^{-1}), GLCM_Imc1 (8.97×10^{-1}), Age (5.50×10^{-3}), M stage (9.23×10^{-1})
	4	first-order_Kurtosis (6.06×10^{-3}), GLRLM_GrayLevelNonUniformityNormalized (1.91×10^{-1}), Age (3.03×10^{-2}), N stage (1.28×10^{-1})
	5	Shape_SurfaceVolumeRatio (7.14×10^{-1}), first-order_Maximum (2.35×10^{-3}), GLCM_Imc1 (3.86), GLSZM_SmallAreaLowGrayLevelEmphasis (2.97), Age (4.46×10^{-2})
SCC_{T3}	1	GLSZM_LargeAreaLowGrayLevelEmphasis (9.77×10^{-5}), Clinical stage (5.66×10^{-2})
	2	GLDM_DependenceVariance (2.51×10^{-2}), GLSZM_ZoneEntropy (1.76), Age (3.55×10^{-2})
	3	GLCM_Correlation (5.37), GLSZM_LargeAreaLowGrayLevelEmphasis (3.08×10^{-5}), M stage (1.23)
	4	GLCM_Correlation (6.21×10^{-1}), GLSZM_LargeAreaLowGrayLevelEmphasis (2.84×10^{-4}), M stage (8.75×10^{-1})
	5	GLSZM_LargeAreaLowGrayLevelEmphasis (8.90×10^{-4}), Clinical stage (1.22×10^{-3})
SCC_{T4}	1	Shape_LeastAxisLength (9.96×10^{-4}), N stage (1.18×10^{-1})
	2	Shape_MajorAxisLength (2.42×10^{-5}), M stage (8.72×10^{-1}), Clinical stage (6.36×10^{-15})
	3	first-order_Energy (1.12×10^{-10}), first-order_TotalEnergy (5.04×10^{-16}), Age (2.09×10^{-2}), M stage (5.39×10^{-1}), Clinical stage (1.16×10^{-14})
	4	Shape_MajorAxisLength (1.90×10^{-5}), Age (1.08×10^{-3}), N stage (2.32×10^{-2})
	5	Shape_MajorAxisLength (3.88×10^{-5}), M stage (3.95×10^{-1}), Clinical stage (7.92×10^{-15})
ADC_{T1}	1	Shape_Flatness (4.90×10^{-1}), first-order_Maximum (3.93×10^{-3}), first-order_Energy (5.20×10^{-10}),

		GLCM_Imc1 (3.16×10^{1}), GLSZM_ZoneEntropy (3.21×10^{-1}), N stage (3.03×10^{-1}), Clinical stage (1.64)
	2	Shape_Flatness (3.72×10^{-1}), Shape_Sphericity (2.51), first-order_Maximum (2.39×10^{-3}), GLCM_Imc1 (1.08×10^{1}), GLSZM_ZoneEntropy (2.95×10^{-1}), Clinical stage (6.26×10^{-1})
	3	Shape_Sphericity (4.30×10^{-1}), first-order_Maximum (1.28×10^{-3}), GLCM_Imc1 (1.85×10^{1}), N stage (1.17×10^{-1}), Clinical stage (4.68×10^{-1})
	4	first-order_Maximum (1.39×10^{-3}), GLCM_Imc1 (4.40), GLSZM_ZoneEntropy (2.12×10^{-1}), Clinical stage (4.84×10^{-1})
	5	Shape_Flatness (5.48×10^{-1}), first-order_Maximum (1.91×10^{-3}), GLCM_Imc1 (7.86), GLSZM_ZoneEntropy (6.36×10^{-1}), Clinical stage (4.90×10^{-1})
ADC_{T2}	1	first-order_Energy (7.38×10^{-11}), first-order_TotalEnergy (2.46×10^{-25}), N stage (1.48×10^{-1})
	2	first-order_Energy (5.70×10^{-11}), N stage (2.66×10^{-1})
	3	GLDM_LargeDependenceLowGrayLevelEmphasis (3.72×10^{-2}), N stage (1.82×10^{-1})
	4	first-order_Energy (6.44×10^{-11}), first-order_TotalEnergy (1.80×10^{-25}), N stage (1.98×10^{-1})
	5	GLDM_LargeDependenceLowGrayLevelEmphasis (2.25×10^{-1}), N stage (1.48×10^{-1})
ADC_{T3}	1	first-order_Kurtosis (5.16×10^{-2}), first-order_Mean (2.16×10^{-4}), Clinical stage (3.11×10^{-1})
	2	Shape_MajorAxisLength (1.43×10^{-2}), M stage (5.09×10^{-3}), Clinical stage (2.72×10^{-1})
	3	Shape_MajorAxisLength (4.60×10^{-3}), first-order_Kurtosis (5.03×10^{-3}), Clinical stage (2.85×10^{-1})
	4	Shape_MajorAxisLength (6.22×10^{-3}), first-order_Kurtosis (1.29×10^{-2}), Clinical stage (8.61×10^{-2})
	5	Shape_MajorAxisLength (1.28×10^{-3}), first-order_Kurtosis (1.06×10^{-2}), Clinical stage (9.09×10^{-2})
ADC_{T4}	1	GLDM_DependenceVariance (5.97×10^{-2}), M stage (3.63×10^{-1}), Clinical stage (2.89×10^{-14})
	2	GLDM_DependenceVariance (4.57×10^{-2}), GLRLM_LongRunEmphasis (7.68×10^{-3}), M stage (1.40)
	3	GLDM_DependenceVariance (1.90×10^{-2}), M stage (1.59×10^{-1}), Clinical stage (7.95×10^{-15})
	4	GLDM_DependenceVariance (7.66×10^{-1}), M stage (1.92×10^{-1}), Clinical stage (1.12×10^{-14})
	5	GLDM_DependenceVariance (4.44×10^{-2}), GLRLM_LongRunEmphasis (2.75×10^{-2}), M stage (9.13×10^{-1})

Abbreviation: SCC = squamous cell carcinoma, ADC = adenocarcinoma,

GLCM = gray-level co-occurrence matrix, GLDM = gray-level dependence matrix,

GLRLM = gray-level run length matrix, GLSZM = gray-level size zone matrix,

NGTDM = neighborhood gray tone difference matrix

Supplementary M: Patient characteristics for the Lung 1 dataset.

**Characteristics**		**Total (n = 287)**
**Age (years: median [range])**		68 [43–92]
**Gender**	Male	207 (72%)
	Female	80 (28%)
**Histological subtype**	Squamous cell carcinoma	82 (29%)
	Adenocarcinoma	27 (9%)
	Large cell carcinoma	92 (32%)
	Not otherwise specified	86 (30%)
**T stage**	1	66 (23%)
	2	114 (40%)
	3	31 (11%)
	4	76 (26%)
**N stage**	0	120 (42%)
	1	13 (4%)
	2	88 (31%)
	3	66 (23%)
**M stage**	0	283 (99%)
	1	4 (1%)
**Clinical stage**	I	74 (26%)
	II	25 (9%)
	III	187 (65%)
	IV	1 (0%)
**Survival time (days: median [range])**		487 [24–2165]
**Survival status**	Survival	99 (34%)
	Death	188 (66%)

Supplementary N: Prognostic performance of the radiomic and combined models in each subgroup with five-fold cross-validation (Lung 1 dataset).

Subgroup	Constructed model		1	2	3	4	5	Overall
**All data (n = 287)**	**Radiomic model**	training	**0.63**	0.64	0.64	0.64	0.60	0.63 ± 0.02
		test	**0.61**	0.62	0.63	0.61	0.60	0.61 ± 0.01
	**Combined model**	training	0.63	0.67	0.68	**0.65**	0.64	0.65 ± 0.02
		test	0.62	0.61	0.60	**0.64**	0.68	0.63 ± 0.03
**SCC_{all} (n = 82)**	**Radiomic model**	training	**0.58**	0.61	0.69	0.61	0.61	0.62 ± 0.04
		test	**0.55**	0.54	0.59	0.52	0.58	0.56 ± 0.03
	**Combined model**	training	0.67	0.62	0.63	**0.66**	0.64	0.65 ± 0.02
		test	0.64	0.51	0.53	**0.63**	0.64	0.59 ± 0.06
**ADC_{all} (n = 27)**	**Radiomic model**	training	0.71	0.64	0.63	**0.67**	0.71	0.67 ± 0.03
		test	0.71	0.63	0.62	**0.65**	0.69	0.66 ± 0.03
	**Combined model**	training	0.66	0.68	**0.71**	0.75	0.73	0.71 ± 0.03
		test	0.70	0.66	**0.71**	0.73	0.73	0.71 ± 0.03

Abbreviation: SCC = squamous cell carcinoma, ADC = adenocarcinoma

Numbers written in bold text: Case closest to the average C-index of the test dataset among the five-fold cross-validation

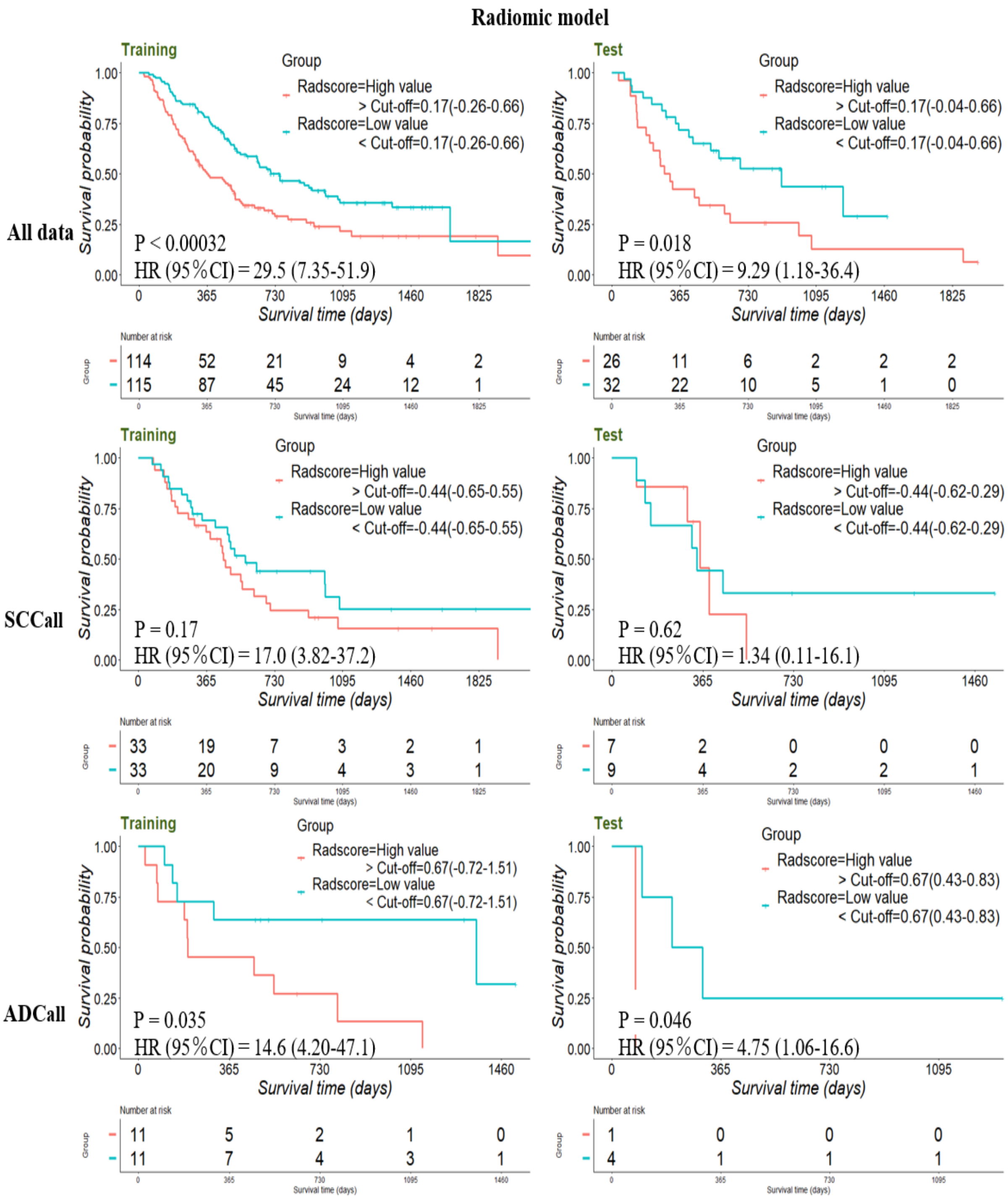

Radiomic model
All data
SCCall
ADCall
Training
Test
Group
Radscore=High value
> Cut-off=0.17(-0.26-0.66)
Radscore=Low value
< Cut-off=0.17(-0.26-0.66)
P < 0.00032
HR (95%CI) = 29.5 (7.35-51.9)
> Cut-off=0.17(-0.04-0.66)
< Cut-off=0.17(-0.04-0.66)
P = 0.018
HR (95%CI) = 9.29 (1.18-36.4)
> Cut-off=-0.44(-0.65-0.55)
< Cut-off=-0.44(-0.65-0.55)
P = 0.17
HR (95%CI) = 17.0 (3.82-37.2)
> Cut-off=-0.44(-0.62-0.29)
< Cut-off=-0.44(-0.62-0.29)
P = 0.62
HR (95%CI) = 1.34 (0.11-16.1)
> Cut-off=0.67(-0.72-1.51)
< Cut-off=0.67(-0.72-1.51)
P = 0.035
HR (95%CI) = 14.6 (4.20-47.1)
> Cut-off=0.67(0.43-0.83)
< Cut-off=0.67(0.43-0.83)
P = 0.046
HR (95%CI) = 4.75 (1.06-16.6)
Survival probability
Survival time (days)
Number at risk
114 52 21 9 4 2
115 87 45 24 12 1
26 11 6 2 2 2
32 22 10 5 1 0
33 19 7 3 2 1
33 20 9 4 3 1
7 2 0 0 0
9 4 2 2 1
11 5 2 1 0
11 7 4 3 1
1 0 0 0
4 1 1 1

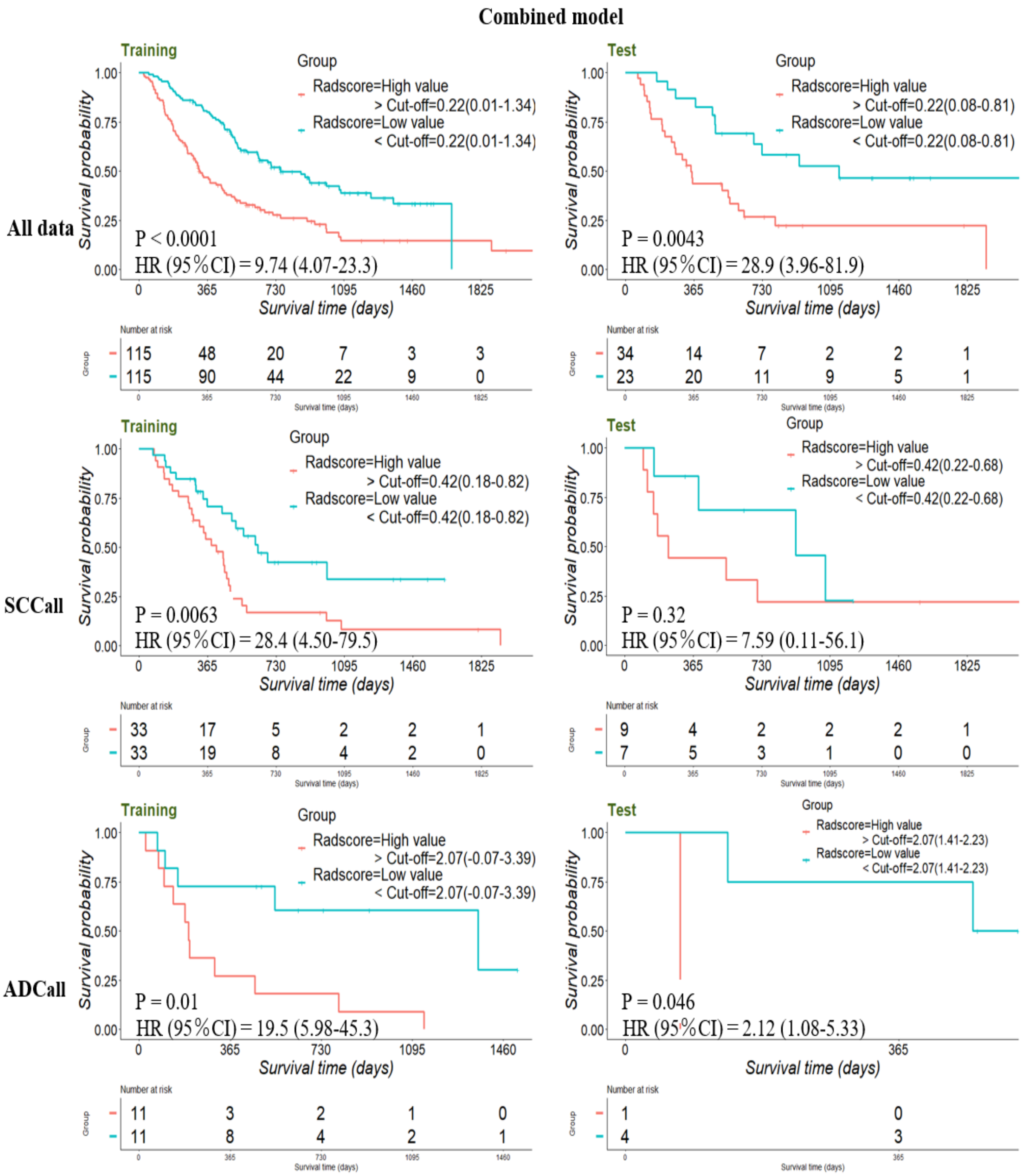

Abbreviation: HR = Hazard ratio, CI = confidence interval

Supplementary O: Kaplan–Meier curves for low- and high-risk groups based on the rad score in the radiomic and combined models for each subgroup (Lung 1 dataset). To avoid complications, the case when it was closest to the mean C-index of the test dataset among the five-fold cross-validation is shown.

Supplementary P: For each analysis group, the features and their coefficients selected in the LASSO Cox regression model in the radiomic and combined models (Lung 1 dataset).

Constructed model	Subgroup	fold	Features (Coefficient)
Radiomic model	All data	1	Shape_MajorAxisLength (2.45×10^{-3}), first-order_Energy (2.57×10^{-12}), first-order_TotalEnergy (8.92×10^{-26}), first-order_Maximum (2.38×10^{-5}), first-order_90Percentile (6.07×10^{-4}), NGTDM_Busyness (4.04×10^{-2})
		2	Shape_MajorAxisLength (3.75×10^{-3}), first-order_Maximum (1.20×10^{-4}), first-order_90Percentile (1.82×10^{-3}), NGTDM_Busyness (5.21×10^{-2})
		3	Shape_MajorAxisLength (5.42×10^{-4}), first-order_Maximum (6.12×10^{-5}), first-order_90Percentile (1.34×10^{-3}), NGTDM_Busyness (2.51×10^{-2})
		4	Shape_MajorAxisLength (9.70×10^{-4}), first-order_Energy (7.68×10^{-12}), first-order_TotalEnergy (4.27×10^{-26}), NGTDM_Busyness (8.07×10^{-2})
		5	Shape_MajorAxisLength (6.00×10^{-3}), Shape_Elongation (1.86×10^{-1}), first-order_90Percentile (2.40×10^{-4}), first-order_Minimum (1.35×10^{-3}), GLSZM_LargeAreaLowGrayLevelEmphasis (3.90×10^{-5})
	SCC_{all}	1	first-order_Minimum (7.07×10^{-4}), NGTDM_Complexity (1.23×10^{-4})
		2	GLSZM_GrayLevelVariance (6.49×10^{-4}), NGTDM_Complexity (1.04×10^{-5})
		3	Shape_MajorAxisLength (2.64×10^{-3}), GLDM_LargeDependenceLowGrayLevelEmphasis (3.96), first-order_Maximum (6.52×10^{-5}), first-order_90Percentile (4.74×10^{-3}), GLSZM_GrayLevelVariance (3.15×10^{-4}), GLSZM_SmallAreaEmphasis (7.67×10^{-1}), GLSZM_SmallAreaLowGrayLevelEmphasis (5.46×10^{1})
		4	first-order_90Percentile (3.27×10^{-4}), GLSZM_GrayLevelVariance (3.71×10^{-3})
		5	first-order_90Percentile (1.58×10^{-4}), GLSZM_GrayLevelVariance (2.02×10^{-3})
	ADC_{all}	1	first-order_90Percentile (7.24×10^{-3}), GLSZM_GrayLevelNonUniformity (1.26×10^{-3})
		2	first-order_90Percentile (6.40×10^{-3}), GLSZM_SizeZoneNonUniformity (3.12×10^{-5})
		3	GLCM_Imc1 (4.45), first-order_Minimum (1.45×10^{-3}), GLRLM_LongRunLowGrayLevelEmphasis (3.29×10^{1})
		4	first-order_Median (9.64×10^{-4}), GLSZM_GrayLevelNonUniformityNormalized (2.52×10^{1})
		5	first-order_90Percentile (1.67×10^{-2}), first-order_Minimum (8.76×10^{-5}), GLSZM_SizeZoneNonUniformity (2.70×10^{-4})
Combined model	All data	1	Shape_MajorAxisLength (3.85×10^{-3}), first-order_Maximum (3.95×10^{-5}), first-order_90Percentile (3.37×10^{-4}), GLSZM_LargeAreaLowGrayLevelEmphasis (2.91×10^{-4}), NGTDM_Busyness (3.83×10^{-2}), Age (1.03×10^{-3}), M stage (1.72×10^{-2})

	2	Shape_MajorAxisLength (8.85×10^{-4}), GLDM_LargeDependenceLowGrayLevelEmphasis (5.37×10^{-2}), first-order_Maximum (8.96×10^{-5}), first-order_90Percentile (1.59×10^{-3}), first-order_Minimum (7.70×10^{-5}), NGTDM_Busyness (7.42×10^{-2}), Age (1.07×10^{-2}), N stage (8.97×10^{-2}), M stage (2.05×10^{-1})
	3	Shape_MajorAxisLength (2.07×10^{-3}), GLDM_LargeDependenceLowGrayLevelEmphasis (9.42×10^{-1}), first-order_Energy (4.82×10^{-11}), first-order_TotalEnergy (3.90×10^{-26}), first-order_90Percentile (1.71×10^{-3}), first-order_Minimum (2.37×10^{-4}), NGTDM_Busyness (2.52×10^{-2}), Age (4.55×10^{-3}), N stage (4.72×10^{-2})
	4	Shape_MajorAxisLength (7.47×10^{-4}), first-order_90Percentile (6.37×10^{-4}), NGTDM_Busyness (5.97×10^{-2}), Age (8.43×10^{-4}), N stage (1.50×10^{-2})
	5	Shape_MajorAxisLength (1.08×10^{-4}), first-order_90Percentile (5.91×10^{-4}), NGTDM_Busyness (5.25×10^{-2}), Age (2.17×10^{-3})
SCC_{all}	1	first-order_Maximum (4.49×10^{-6}), first-order_90Percentile (4.85×10^{-4}), N stage (3.06×10^{-1}), Clinical stage (4.55×10^{-3})
	2	first-order_Maximum (2.47×10^{-5}), GLSZM_GrayLevelVariance (1.17×10^{-3}), Age (8.13×10^{-2}), Clinical stage (2.09×10^{-2})
	3	GLDM_LargeDependenceLowGrayLevelEmphasis/gldm (1.16×10^{-1}), first-order_90Percentile (1.54×10^{-3}), GLSZM_GrayLevelVariance (4.94×10^{-3}), GLSZM_SmallAreaLowGrayLevelEmphasis (2.07), N stage (5.55×10^{-3})
	4	first-order_90Percentile (1.13×10^{-3}), GLSZM_GrayLevelVariance (1.85×10^{-3}), GLSZM_SmallAreaLowGrayLevelEmphasis (2.88×10^{1}), N stage (9.01×10^{-2})
	5	First-order_Maximum (7.44×10^{-5}), first-order_90Percentile (9.43×10^{-4}), N stage (4.84×10^{-2})
ADC_{all}	1	first-order_Maximum (1.08×10^{-4}), GLRLM_LongRunHighGrayLevelEmphasis (2.83×10^{-5}), first-order_90Percentile (2.63×10^{-3}), GLSZM_SizeZoneNonUniformity (1.03×10^{-5}), N stage (5.09×10^{-2}), Clinical stage (4.77×10^{-2})
	2	Shape_Flatness (1.24), first-order_90Percentile (6.20×10^{-3}), GLSZM_SizeZoneNonUniformityNormalized (3.21), GLSZM_SizeZoneNonUniformity (3.42×10^{-4}), T stage (1.06×10^{-1}), Clinical stage (1.21×10^{-1})
	3	GLCM_Imc1 (5.58), first-order_Minimum (2.12×10^{-3}), N stage (7.67×10^{-1}), Clinical stage (4.30×10^{-3})
	4	GLCM_MaximumProbability (2.08), first-order_90Percentile (1.22×10^{-2}), Clinical stage (2.64×10^{-1})

5	Shape_Flatness (1.07), first-order_Median (6.24×10^{-4}), first-order_90Percentile (9.40×10^{-3}), GLSZM_SizeZoneNonUniformity (3.46×10^{-4}), GLSZM_SmallAreaEmphasis (2.26), Age (3.04×10^{-2}), Clinical stage (2.97×10^{-1})

Abbreviation: SCC = squamous cell carcinoma, ADC = adenocarcinoma,

GLCM = gray-level co-occurrence matrix, GLDM = gray-level dependence matrix,

GLRLM = gray-level run length matrix, GLSZM = gray-level size zone matrix,

NGTDM = neighborhood gray tone difference matrix
